# The complex impact of China’s science and technology talent policies on key core technologies R&D

**DOI:** 10.1371/journal.pone.0324587

**Published:** 2025-05-29

**Authors:** Pu Miao, Xiaoyan Zhang, Ning Zhang

**Affiliations:** 1 School of Economics and Management, Shanghai Maritime University, Shanghai, China; 2 School of Tourism and Wellness, Changzhou Vocational Institute of Industry Technology, Changzhou, Jiangsu, China; 3 School of Digital Business, Changzhou Vocational Institute of Industry Technology, Changzhou, Jiangsu, China; Wuhan University, CHINA

## Abstract

Key core technologies have become the focal point of global technological competition. For latecomer countries, formulating effective science and technology talent policies to stimulate researchers’ motivation and innovation potential is crucial for mastering these key technologies. This study uses a sample of 1363 publicly listed enterprises in China from 2011 to 2023 to investigate the impact of science and technology talent policies on key core technologies R&D in enterprises. The findings are as follows: (1) Science and technology talent policies significantly promote key core technologies R&D; however, their impact is stronger on non-key core technologies R&D and utility model R&D than on key core technologies R&D. Further study found that non-key core technologies R&D and utility model R&D have a crowding-out effect on key core technologies R&D to a certain extent, and the effect of the former is stronger than that of the latter. (2) As the intensity of policy support increases, its impact on key core technologies R&D also strengthens. Moreover, policies related to talent subsidies, innovation incentives, talent introduction, and high-level talent innovation support all facilitate key core technologies R&D, although the effects vary across these policy types. Moreover, there is no interaction between talent subsidies and innovation incentives, or between talent subsidies and high-level talent innovation support, but there is an offsetting effect between the other policies. (3) The positive impact of science and technology talent policies exhibits a time lag, generally ranging from 4 to 5 years. During this lag period, the effect on the number of patent declines by 8.475% to 28.283%, while the impact on the number of citations of patents decreases by 55.696% to 73.214%. (4) The significant promotional effect of science and technology talent policies is most pronounced in non-state-owned enterprises and those with high R&D investment, but such policies do not have a notable impact on state-owned enterprises or those with low R&D investment. The study demonstrates that latecomer countries can effectively promote key core technologies R&D by designing science and technology talent policies. Based on these findings, recommendations are made to enhance the support intensity of talent policies, optimize the synergistic effects of these policies, extend the duration of policy support, and implement differentiated measures for policy execution.

## 1. Introduction

In today’s world, science and technology occupy a central position in global competition, and mastering key core technologies is one of the hallmarks of gaining an advantage in technological competition. Key core technologies are those positioned at the core of technological systems, characterized by high complexity and substantial research and development (R&D) challenges compared to general technologies. Due to the significant investment, high risks, and long development cycles involved, many enterprises face difficulties in developing key core technologies. Therefore, how to effectively promote R&D in these technologies has become a key concern for countries worldwide. The resources invested in key core technologies R&D include R&D funding and human capital. Existing studies have focused on the impact of R&D funding on innovation outputs, including various funding sources such as self-financed capital [1], government subsidies [2], tax reductions [3], science and technology innovation programs [4], and venture capital [5]. However, these studies fail to recognize the fact that talent, as the implementers of research, plays a crucial role. Given the complexity of key core technologies, fully stimulating the R&D motivation and innovative potential of talent is essential for achieving successful technological breakthroughs. In this regard, the Chinese government has implemented various talent policies for technology-intensive emerging industries, aiming to provide an optimal working and living environment for talent, thereby enabling them to fully devote themselves to research and enhancing R&D performance. However, little is known about whether and how these policies influence key core technologies R&D in enterprises. This study seeks to address this gap, with the aim of contributing to the theoretical understanding of the impact of government policies on technological innovation, specifically examining how science and technology talent policies in latecomer countries influence key core technologies R&D. In doing so, the study seeks to provide new insights to help latecomer countries drive key core technologies R&D.

Over the past three years, research on the impact of policies on technological innovation has garnered increasing attention. These studies generally focus on the effects of specific types of policies, particularly environmental policies, as well as policies promoting innovation in fields such as new energy vehicles, macro-industrial policies, intellectual property policies, and entrepreneurship support policies, while also considering the uncertainties associated with these policies. Overall, these studies have demonstrated that such policies can foster technological innovation, although their effects are often heterogeneous and nonlinear. For example, studies have found that low-carbon pilot policies [6], carbon emission trading policies [7], green fiscal policies [8], green financial policies [9], green credit policies [10], macro environmental policies [11], renewable energy policies [12], and “Broadband China” policies [13] have had a consistent positive effect on technological innovation, especially in low-carbon and green technology innovation. Similar findings have been reported for policies aimed at promoting technological innovation in the new energy vehicle sector, such as government support policies, regulatory policies [14], fiscal subsidies [2], tax incentives [3], tax exemption policy for car purchases [15], and policy for pilot cities of new energy vehicles [16]. However, these policies are found to significantly impact the quantity of innovation but have limited effects on the quality of technological innovations, suggesting that their influence may be short-term, or that enterprises may engage in “pseudo-innovation” to gain policy support.

Further research has also revealed that the heterogeneous impacts of policies are widespread. A study on EU innovation policies found that such policies have a short-term positive effect on technological innovation, especially for financially sound and more internationalized enterprises, while their effects on small and medium-sized enterprises are negligible [17]. Government subsidies, tax credits, and loan support policies were found to have an inverted U-shaped relationship with technological innovation, indicating that excessive policy support may stifle innovation [18]. Green credit policies have a negative impact on the innovation output of pollution-intensive enterprises but significantly promote innovation in clean energy enterprises [10,19]. These studies underscore the complexity of policy effects on technological innovation. Additionally, the relationship between policy uncertainty and technological innovation is also complex. Research has found that economic policy uncertainty promotes the quantity of innovation but inhibits its quality. Increased uncertainty heightens enterprise risks, prompting enterprises to quickly invest in innovation to capture future growth opportunities [20]. However, some studies suggest that low levels of policy uncertainty can stimulate technological innovation [21]. We observe that most existing studies have focused on policies related to funding, administrative management, and public services, neglecting the role of talent policies in stimulating the innovation potential of scientific personnel. This gap in research limits our understanding of how to fundamentally drive technological innovation. Moreover, existing studies have largely overlooked key core technologies R&D. Unlike general technologies, key core technologies have strategic value and are a focal point in the technological competition between developed and developing countries. Therefore, studying the impact of science and technology talent policies on key core technologies R&D is of critical importance.

In this study, science and technology talent policies refer to a series of measures designed to promote technological R&D by providing favorable incentives in areas such as financial rewards, career advancement, spouse employment, children’s education, housing subsidies, medical benefits, and living services, aiming to stimulate R&D motivation and innovation potential of talent. The Chinese government has explicitly outlined the need to promote breakthroughs in key core technologies within strategic emerging industries in its “14th Five-Year Plan”. In line with this, local governments, guided by central policy and performance evaluation constraints, have implemented various talent policies to encourage technological breakthroughs. According to the *China Regional Science and Technology Innovation Evaluation Report (2023)* and the *China Science and Technology Talent Development Report (2022)*, all 31 provincial-level administrative regions in China (excluding Hong Kong, Macau, and Taiwan) have issued science and technology talent policies. In the economically developed regions of Jiangsu, Zhejiang, and Shanghai, every city at the district level has published talent policies, with some even issuing policies at the county level. How do these talent policies impact key core technologies R&D? This question has not been addressed in existing studies. From a theoretical perspective, science and technology talent policies are likely to have a positive impact on key core technologies R&D. First, financial rewards in these policies can alleviate the funding constraints faced by talent in developing key core technologies. Second, other measures in these policies provide a stable working and living environment, thereby enhancing the R&D motivation of talent and stimulating their innovative potential.

Based on this theoretical expectation, this study empirically examines the impact of science and technology talent policies on key core technologies R&D. Specifically, we address three questions: First, do science and technology talent policies promote key core technologies R&D, and is there heterogeneity in their effects? Second, do different levels of policy support and policy types have varying effects on key core technologies R&D, and what are the differences? Third, do science and technology talent policies have delayed effects, and if so, what patterns do they show? To address these questions, we conduct empirical research using a sample of 1363 publicly listed enterprises in China from 2011 to 2023. The results of our study are as follows: First, science and technology talent policies have a significant positive impact on key core technologies R&D, but they have a stronger impact on non-key core technologies R&D and utility model R&D. Second, the stronger the policy support, the greater the positive effect on key core technologies R&D. Among various policies, talent subsidies, innovation incentives, talent introduction, and high-level talent innovation support all significantly promote key core technologies R&D, although high-level talent innovation support has a relatively smaller effect. Third, the positive impact of science and technology talent policies on key core technologies R&D is delayed, remaining effective 4–5 years after policy implementation. As the lag period increases, the impact of the policies on the number of patent decreases, ranging from 8.475% to 28.283%, while the impact on the number of citations of patents decreases between 55.696% and 73.214%. Fourth, the heterogeneous effects of science and technology talent policies show that these policies significantly promote key core technologies R&D in non-state-owned enterprises and high R&D investment enterprises, but have no significant impact on state-owned enterprises or those with low R&D investment. These research findings suggest that science and technology talent policies can effectively promote key core technologies R&D. However, the impact of these policies varies across different levels of policy support, types of policies, and lag periods. Moreover, the effect of the policies exhibits heterogeneity depending on the ownership structure of the enterprises and the intensity of their R&D investments.

These findings provide empirical evidence on the role of science and technology talent policies in promoting key core technologies R&D, filling gaps in existing policy research, and extending our understanding of policy heterogeneity effects. Our contributions to the existing literature are as follows: First, unlike previous studies that only focus on the impact of policies related to funding, administrative management, and public services on technological innovation, we focus on the unique role of science and technology talent policies in stimulating the R&D motivation and innovative potential of talent. This is the first study to demonstrate the significant impact of talent policies on key core technologies R&D, even though such policies also have a stronger effect on non-key core technologies R&D and utility model R&D. This finding provides new perspectives and empirical evidence for latecomer countries to promote key core technologies R&D through the formulation of feasible science and technology talent policies. Second, unlike previous studies that only focus on the effects of similar policies on different target groups, we investigate the heterogeneous impact of science and technology talent policies themselves on key core technologies R&D. Our study shows that as the intensity of policy support increases, its positive effect on key core technologies R&D also increases, with varying effects across different types of policies. This finding provides empirical support for the development of science and technology talent policies with differentiated levels of support and policy types tailored to the varying challenges of technological innovation activities, thereby expanding our understanding of the heterogeneous impacts of policy interventions. Third, unlike previous studies that only reveal the lagged effects of policies on technological innovation, we delve deeper into the attenuation pattern of these effects. We find that the impact of science and technology talent policies on the number of citations of patents decreases more rapidly over time, while the decline in their effect on the number of patents is relatively slower. This finding allows for a more nuanced understanding of how policies influence both the quantity and quality of patents, offering new insights into existing studies on the impact of policies on technological innovation.

## 2. Theoretical analysis and research hypotheses

In this section, we start by exploring the characteristics of key core technologies R&D, discussing the role of enterprises and the challenges they face in this area. We then analyze how science and technology talent policies can help enterprises address these challenges and, based on this, propose the research hypotheses for this study.

Compared to general technologies, key core technologies have two distinct characteristics: First, key core technologies are monopolized by a few leading enterprises globally. These enterprises leverage their technological advantages to influence global industrial and innovation chains, yielding substantial profit returns. Such technologies are tightly controlled by these enterprises, making it difficult for latecomers to access them through the market. Second, key core technologies are highly complex, consisting of multiple specialized technologies that form a rigorous technical system. They are often developed through collaboration among enterprises with expertise in specific subfields. It is extremely difficult for a single enterprise, relying solely on its own resources, to make breakthroughs. Due to these two characteristics, the resource investment required for key core technologies R&D tends to be high, with long development cycles and high risks. Therefore, enterprises with abundant scientific and technological resources and the ability to quickly integrate various resources will play a crucial role in key core technologies R&D.

Large enterprises possess significant advantages in this regard, which cannot be matched by medium and small-sized enterprises. Large enterprises have the following advantages in integrating scientific and technological resources: They can achieve economies of scale in scientific research, thereby improving the efficiency of resource utilization and reducing R&D costs [22]. They have abundant technological resources and strong capital strength, enabling them to rapidly acquire funding, talent, and market resources through mergers, acquisitions, and collaborative R&D [22]. They have accumulated expertise in different technological fields and possess well-established R&D systems, enabling them to create synergies by integrating internal and external technological resources [23]. They also have significant influence on local economies, which allows them to obtain better government support and financing channels, enhancing their capacity for technological breakthroughs [24,25]. Thus, large enterprises with stronger technological resource integration capabilities play an important role in key core technologies R&D.

Although large enterprises are an important force in key core technologies R&D, they also face challenges in integrating technological resources and stimulating resource effectiveness, particularly in the integration and incentivization of talent. First, attracting and retaining high-level talent is a critical issue. While large enterprises usually possess superior resources and infrastructure, attracting high-level talent and ensuring their long-term retention remains one of the core bottlenecks in technological innovation, particularly against the backdrop of increasing global competition and rapid technological change. Second, interdisciplinary and cross-departmental collaboration barriers severely hinder R&D efficiency. Breakthroughs in key core technologies require close cooperation among experts from various disciplines. However, internal organizational barriers and poor information flow often impede effective collaboration, slowing the R&D process and reducing innovation outcomes. Third, existing incentive mechanisms have limitations in motivating talent’s innovative engagement. Although large enterprises can offer higher salaries, traditional compensation-based incentives often fail to meet the needs of talent for innovation space, research support, and career development, which leads to the underutilization of their potential. Fourth, maintaining the long-term innovative drive of talent is an ongoing challenge. Key core technologies R&D requires long-term investment and technical accumulation. However, large enterprises often face shortcomings in providing long-term incentives and support for research personnel, which makes it difficult to sustain their innovation drive, thus affecting the continuity and depth of technological development. These challenges represent the talent bottlenecks faced by large enterprises in key core technologies R&D.

Due to the challenges that large enterprises face in integrating talent and stimulating their innovative effectiveness during key core technologies R&D, current science and technology talent policies provide multi-dimensional support that effectively promotes key core technologies R&D.

First, to address the issue of attracting and retaining high-level talent, the government has established policies such as the “Thousand Talents Plan,” “Ten Thousand Talents Plan,” and the “Innovation Talent Promotion Plan,” offering support in areas such as generous salaries, research funding, professional title promotion, housing security, and children’s education. These policies significantly enhance enterprises’ ability to attract high-level talent. Through policy-driven funding support, talent cultivation programs, and project funding, they also ensure workforce stability, providing a talent guarantee for enterprises undertaking long-term, high-risk, and high-investment key core technologies R&D. According to human capital theory [26], the attraction of high-level talent is not only based on salary but also on research resources, career development opportunities, and life security. These external incentives significantly enhance the innovation motivation and job satisfaction of talent. Government-provided stable research funding, professional title advancement opportunities, and social welfare measures can effectively improve talent satisfaction [27], thereby enhancing their loyalty to the enterprise [28]. They also improve job satisfaction and organizational commitment [29]. It is clear that through long-term financial support and career development opportunities, government policies not only attract high-level talent but also strengthen talent’s long-term reliance on enterprises, thus enhancing the technological R&D capacity of enterprises. Therefore, science and technology talent policies, through various measures, improve the talent supply environment for enterprises, attract and retain talent, ensure the stability of R&D teams, and contribute to the enhancement of enterprises’ key core technologies R&D capabilities.

Second, to address the challenges of interdisciplinary and cross-departmental collaborative innovation, talent policies guide enterprises in building interdisciplinary R&D teams and provide funding support for cross-field cooperation, effectively overcoming barriers to collaboration in technological development. Policies such as the “Research Team Construction Policy” and the “Industry-Academia-Research Cooperation Policy”, introduced by both local and central governments, not only offer financial assistance to enterprises but also promote the formation of interdisciplinary and cross-field R&D teams, facilitating the integration of talent from various disciplines. This policy framework helps overcome the limitations of individual disciplines and departments, fosters better information flow, and encourages collaborative innovation among researchers, ultimately improving R&D efficiency [30,31]. Interdisciplinary collaboration can significantly enhance the depth and breadth of technological innovation. Particularly when facing complex technological systems, professionals from different fields can facilitate knowledge diffusion through collaborative research networks [32], thus advancing technological progress. Moreover, enterprises often face higher transaction costs and opportunistic behaviors during interdisciplinary cooperation, but through financial support and policy incentives, the government can effectively reduce these costs, promote resource sharing, and foster collaborative innovation, thereby improving R&D efficiency [33]. Therefore, current science and technology talent policies, by supporting enterprises in carrying out interdisciplinary and cross-departmental collaborative innovation, encourage the flow of technical knowledge and enhance its spillover effects, thus contributing to key core technologies R&D.

Third, to address the issue of stimulating talent’s innovation motivation, current talent policies provide innovative incentive measures aimed at boosting the creativity of talent. The “Innovation Incentive Policies” in these programs, through funding support for research projects, professional title promotions, and research awards, stimulate the initiative and creativity of talent. Particularly in the “High-Level Talent Innovation Support Policy,” support is extended to academicians, PhDs, leading scientific and technological figures, and overseas high-level talents. Not only does this provide financial support, but it also offers talent rich career development paths and ample technological innovation space. According to innovation motivation theory, external incentive measures can strengthen an individual’s innovation drive and encourage greater effort in technological innovation activities [34]. Specifically, research funding, title promotions, and rewards can improve job satisfaction [29], thereby increasing the enterprise’s innovative output [35]. Furthermore, comprehensive incentive measures, while enhancing the innovation motivation of talent, can also reduce perceived innovation risks [36], thus encouraging them to engage in high-risk technological challenges. Therefore, by providing diverse science and technology talent incentive policies, the government can effectively stimulate the innovation vitality of talent and drive enterprises to carry out key core technologies R&D.

Fourth, to address the issue of maintaining long-term innovation motivation for talent, talent policies provide continuous research support, long-term career development plans, and superior social security measures, effectively solving the problem of insufficient motivation in long-term technological R&D. The government, through programs such as the “Innovation Talent Support Plan” and special research funds, offers stable research funding, long-term career plans, and abundant research resources, helping enterprises ensure the long-term, stable engagement of talent [37], thereby enhancing talent’s motivation and innovation drive in long-term technological R&D. Innovation incentive theory suggests that innovation rewards, superior social security, and other incentive factors can improve the long-term innovation performance of enterprise talent [38,39]. The stable research funding and technological fund projects provided by the government help mitigate the negative effects of innovation uncertainty and stimulate long-term innovation investment by talent [40]. Such stable support also plays a role in long-term innovation development plans, assisting talent in defining their career development and gradually clarifying their research directions, thus unlocking their research potential. For example, government-funded research projects can reduce the negative impact of external economic fluctuations on R&D investment [41], thereby providing reliable funding security for talent. Moreover, long-term technological fund projects can help enterprises cultivate core talent dedicated to technical research, further enhancing the enterprises’ innovation capability [4]. Therefore, talent policies, by providing continuous research funding, career development opportunities, and social security, can effectively maintain the innovation drive of talent, ensuring their long-term commitment to core technology R&D.

In summary, we believe that science and technology talent policies can help enterprises address the talent challenges they encounter in the process of key core technologies R&D through the aforementioned approaches, thereby contributing to the improvement of their performance in key core technologies R&D. Based on this, the research hypothesis of this study is as follows: Compared to enterprises that do not receive support from science and technology talent policies, enterprises that receive such support can significantly improve their performance in key core technologies R&D.

## 3. Research design

### 3.1. Background of science and technology talent policies

**Fig 1:**
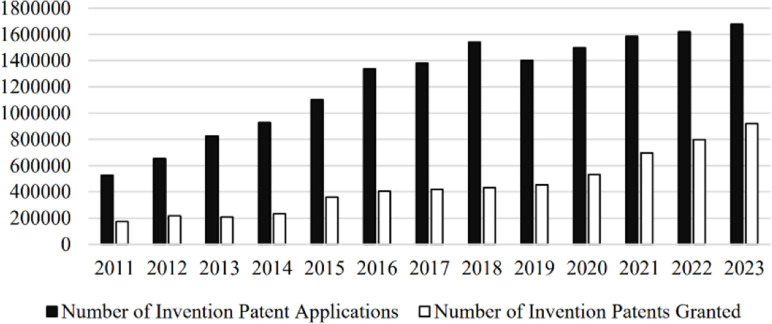
Invention patent applications and grants in China.

Since 2011, China’s technological innovation capabilities have significantly improved. Fig 1 illustrates the number of invention patent applications and grants in China from 2011 to 2023, demonstrating a consistent upward trend for both indicators. Specifically, the number of invention patent applications grew from 526412 in 2011 to 1677701 in 2023, a 219% increase, while the number of granted invention patents rose from 172113 in 2011 to 920,797 in 2023, reflecting a growth of 435%. This change is attributed not only to enterprises’ efforts to establish themselves in the market but also to a series of technological innovation policies implemented by the Chinese government. The implementation of these policies has not only promoted progress in technological R&D but also strengthened China’s competitiveness in global technological innovation. Since 2011, several important national-level technological innovation strategies have been introduced in China, including the Innovation-driven Development Strategy (2012), Made in China 2025 (2015), Science and Technology Innovation 2030-Major Project (2016), and the 14th Five-Year Plan (2021). These strategies have injected strong momentum into China’s technological innovation, laying the foundation for talent policy formulation. Under the guidance of these policies, in 2022, the Chinese government explicitly proposed the integrated implementation of the strategy to revitalize the country through science and education, the innovation-driven development strategy, and the talent-strengthening strategy, with a focus on advancing education, scientific innovation, and talent cultivation, thereby strengthening the strategic position of talent. Particularly in recent years, as international competition has become increasingly fierce, mastering key core technologies has become crucial for China to enhance its industrial competitiveness and international influence. Against this backdrop, the role of talent has become increasingly prominent, and how to effectively cultivate, introduce, and retain high-level talent has become an important component of national strategy.

To address this challenge, various talent policies have been implemented by different levels of government in China. Representative examples include the “Thousand Talents Program”, “Ten Thousand Talents Program”, “Innovation Talent Promotion Program”, “Local Talent Introduction Policies”, and “Talent Housing Security and Preferential Policies”. However, different levels of government in China formulate science and technology talent policies based on the regional economic development levels and the supply-demand dynamics of the human capital market. As a result, these policies vary in name, type, and terms. Based on the content provided by the talent policies for the sample enterprises, the talent policies received by the sample enterprises can be classified into four categories: talent subsidies, innovation incentives, talent introduction, and high-level talent innovation support. Talent subsidy policies primarily focus on providing financial assistance for daily living expenses and salaries without directly supporting technological innovation. Innovation incentive policies encourage talent to engage in technological innovation by providing financial support and title promotions, with a primary focus on fostering innovation. Talent introduction policies encourage enterprises to recruit talent through various channels, offering corresponding introduction subsidies. High-level talent innovation support policies mainly support high-level talents, such as academicians, doctors, scientific leaders, and high-level overseas talent, in conducting technological innovation, with forms of support such as financial backing, title promotion, and preferential policies for family education and healthcare. From the perspective of policy provisions, the specific content of these policies varies greatly, but they generally involve support for research funding, living subsidies, job and title promotions, social security and educational benefits for children, housing security and preferences, research team construction, and the transformation and benefit-sharing of scientific achievements. These policy elements complement each other and work together, although the emphasis of each policy differs. For example, talent subsidy policies emphasize providing subsidies to improve the daily salary and treatment of talent; innovation incentive policies focus on supporting talent in terms of technological innovation; talent introduction policies stress attracting talent through treatment and research-related preferential policies; and high-level talent innovation support policies focus on stimulating the innovative potential of high-level talent.

The implementation of these policies is usually designed and issued by the Chinese Ministry of Science and Technology (MOST), Ministry of Education (MOE), Ministry of Human Resources and Social Security (MOHRSS), Ministry of Industry and Information Technology (MIIT), State-owned Assets Supervision and Administration Commission of the State Council (SASAC), and National Natural Science Foundation of China (NSFC) as the core driving departments in conjunction with other ministries, commissions, and local governments, in accordance with the national strategy for science and technology innovation. First, the government formulates specific talent policies according to national development needs and local characteristics, and releases relevant notices through official channels to clarify the direction of support, funding criteria and application conditions. After the release of the policy, all relevant government departments will organize the declaration of talent policy and project establishment, and enterprises need to submit application materials according to the policy requirements. These applications usually involve detailed descriptions of technological innovation and talent needs. Government departments will conduct rigorous review and assessment of the application projects, and decide whether to fund and support them through procedures such as expert review and panel discussion. Successful projects will enter the implementation stage, with the relevant funds allocated according to the project plan. The government continues to track the progress of the projects and conduct acceptance and assessment upon completion. Throughout the process, the government ensures that the policies can be effectively implemented and promote technological innovation through a fine-grained policy design and monitoring mechanism.

The process for an enterprise to obtain support for science and technology talent policies usually consists of four steps: policy declaration, qualification review, fund allocation and follow-up assessment. First, enterprises prepare and submit declaration materials based on the policy information released by the government, which usually include proof of the enterprise’s R&D investment, talent employment contracts, and innovation project plans. Enterprises need to ensure that their declaration materials comply with the policy requirements and submit targeted project plans and relevant certificates according to different policy categories. Next, the government audits the declaration materials, assessing the enterprise’s industry status, technology level, talent structure and R&D capability, and some local governments will also organize expert reviews to ensure that the project meets the policy requirements. After passing the audit, the enterprise will start to receive government funding, and some policies may adopt a phased allocation or “implement first, subsidize later” approach to provide financial support after the enterprise completes the established R&D objectives. Finally, enterprises are required to submit regular R&D progress reports in accordance with the policy requirements, accept the government’s performance evaluation to ensure that the use of funds is in line with the expected goals, and complete the project acceptance in accordance with the regulations.

The above process is illustrated by the Qiming (QM) Program, which was initiated and coordinated by the MIIT. The program aims to promote key core technologies breakthroughs by relying on local state-owned enterprises, private enterprises and branches of central enterprises to introduce overseas high-level talents. The entire implementation process includes policy release, declaration acceptance, evaluation, fund allocation and assessment. First of all, the central and local governments release the policy and set up special funds. Local enterprises conduct internal screening, matching and signing of intent agreements according to the talent requirements, and prepare and submit relevant materials, such as enterprise qualifications, talent qualifications, R&D achievements and project plans. The local government conducts a preliminary review of the declaration materials and recommends them to the MIIT. The department conducts a centralized review of all the declaration materials, determines the selected list, and provides support such as financial subsidies, scientific research funds and salary subsidies to the selected talents. After the implementation of the project, the MIIT and its commissioned units conduct an annual assessment based on the performance of the enterprises to ensure the continuity of the financial support, while the enterprises are required to complete the project in accordance with the regulations and undergo performance evaluation.

The Chinese government’s macro science and technology talent policies, through precise design and implementation, aims to promote the national science and technology innovation strategy while providing a stable support framework for enterprises. The support that enterprises receive under this framework can effectively assist in the R&D of key core technologies and the introduction of high-level talent. However, the success of enterprises in obtaining these policy supports depends on the declaration materials, technology innovation plans and R&D capabilities that meet the policy requirements. After an enterprise passes the audit and receives support, the government’s fund allocation and follow-up assessment mechanism ensures the effective implementation of the project. Through such policy implementation and enterprise cooperation, the government’s macro-policies and enterprises’ micro-needs are well matched, promoting the development of science and technology innovation.

### 3.2. Model design

Since enterprises receive support from science and technology talent policies at different times, a multi-period difference-in-differences (DID) model is constructed to examine the impact of science and technology talent policies on key core technologies R&D. The model is structured as follows:


$$KCT_{it}=\alpha +\beta TP_{it}+\gamma_{i}\sum {\mathrm{\backslash nolimits}}_{m=1}^{n}CV_{m}+industry_{i}+year_{t}+\varepsilon_{it}$$ (1)

where KCT denotes key core technologies R&D, TP denotes science and technology talent policies, CV denotes a series of control variables (detailed below). In the model, i denotes the individual enterprise, t denotes time, year_t_ denotes time fixed effects, and industry_i_ denotes industry fixed effects. Where α, β, and γ are the parameters to be estimated, and ∊ is the error term. Since all variables in this study are measured at the enterprise level, we draw on the work of Bollen and Brand [42] to incorporate industry-level fixed effects into the model, in order to mitigate potential collinearity issues.

### 3.3. Variables

#### 3.3.1. Dependent variable.

The dependent variable is key core technologies R&D (KCT). Key core technologies refer to technologies that occupy a critical position in a related field’s technological system. They are more complex, have longer R&D cycles, and involve higher risks compared to other technologies. These technologies are fundamental, original, and have broad influence. There is no globally unified standard for identifying key core technologies. From a temporal perspective, technologies evolve continuously. Technologies that were once considered key core technologies may no longer be regarded as such as knowledge diffuses over time. Furthermore, in different technological fields, the complexity of the technological system varies, and general indicators might not effectively identify key core technologies across all fields, as their significance can only be assessed within their specific domains. Based on the above analysis, it can be concluded that identifying a universally applicable and fixed standard for measuring key core technologies is challenging.

However, to explore the impact of talent policies on key core technologies R&D, an appropriate benchmark needs to be established to determine the scope of key core technologies. According to existing studies, key core technologies can be reflected in high-level invention patents. Inventions are legally protected by patents and often represent technological originality and advancement. The patent review process is strict, requiring innovation that not only involves technological breakthroughs but also demonstrates feasibility. Invention patents objectively reflect the level of innovation and technological advancement. Moreover, the patent system ensures that technological information is detailed and publicly available. From the perspective of information accessibility and technological level, invention patents are a good measure for assessing key core technologies. Thus, in this study, invention patents are chosen as the data source to define key core technologies. The number of citations of patents are often regarded as an important indicator of patent quality because the number of citations reflects the innovation and impact of the technology. Studies show that the number of citations of patents represents their influence and value in the technological field. A patent’s citation frequency directly reflects its contribution to advancing other research and innovations. Patents with higher citation counts are typically seen as high-quality technological achievements [43]. Therefore, the number of citations of patents is one of the key indicators for assessing its quality.

According to the research by Bronwyn H Hall [44], he found that for companies with the number of citations of patents below the median, market value does not change significantly regardless of the extent of this shortfall. However, for companies with the number of citations of patents above the median, their market value increases significantly. For instance, if the number of citations of patents is between 7 and 10, market value rises by 10%; between 11 and 20, market value increases by 35%; and if the number of citations of patents exceeds 20, the company’s market value is 54% higher than expected. His data indicates that patent citation counts are highly skewed: of one million patents, a quarter have not been cited, 150000 have only been cited once, 125000 twice, and only four have been cited more than 200 times. Although the paper does not directly disclose the citation distribution of other patents, it is evident that more than 50% of all patents have been cited fewer than twice. This indicates that patents with greater value or those that have a significant impact on other patents constitute a very small proportion of the total number of patents. While no study directly specifies that patents cited in the top 10%, 5%, or 1% represent key core patents, it is clear that highly cited patents typically include a significant number of key core patents. Both the OECD (2011) and NSF (2014) identify patents in the top 5% as part of the “high-level” patent group. Therefore, this study first uses the top 5% citation benchmark to define key core technologies in baseline regressions. However, to more comprehensively analyze key core technologies R&D, we will also employ patent citation data from the top 1%, 10%, 15%, 20%, and 25% for robustness checks and perform quantile regressions at the 1%, 5%, 10%, 15%, 20%, and 25% citation quantiles. Therefore, drawing from Bronwyn H. Hall’s research [44], we use the number of invention patents and the number of citations of invention patents as indicators of key core technologies R&D. The following explains these two indicators.

(1) Number of Invention Patents (KCT1)

The number of invention patents is represented by the number of granted patents, as granted patents have passed the patent office’s review, indicating their innovation relative to existing patents. Additionally, some enterprises apply for a large number of patents to create patent barriers and prevent imitation, which may lead to inflated the number of patent applications. To avoid this, we use the number of granted patents instead of the number of applications. Since the focus of this study is on the impact of science and technology talent policies on key core technologies R&D, and the granting of patents lags behind patent applications, it does not reflect the R&D output of the application year in a timely manner. Therefore, we match granted invention patents to their application years to reflect the R&D output of the application year.

To capture key core technologies R&D, we rank the processed the number of citations of patents (the method is explained below) and select patents ranked in the top 1%, 5%, 10%, 15%, 20%, and 25%. For each sample enterprise, the number of granted patents at these six citation ranks is constructed as the indicator KCT1.

(2) Number of Citations of Invention Patents (KCT2)

The number of citations of patents represent the quality, status, and influence of a patent within its technological field. Hence, key core patents should have a higher number of citations. However, the number of citations of patents suffer from truncation issues, so we address this by adjusting for both the temporal and technological domain aspects, aiming to obtain more accurate citation counts. The adjustment for the number of citations is as follows:


$$KCT2=APCN_{ilt}/APCN_{lt}$$ (2)

where APCN_ilt_ denotes the average number of citations of patents for enterprise i in technological domain l in year t, and APCN_lt_ denotes the average number of citations of patents for all sample enterprises in technological domain l in year t. We use the internationally recognized IPC classification system for patents, while taking into account operability, and select patent section as the criterion for defining technological fields.

In order to compare the impact of science and technology talent policies on non-key core technologies R&D and utility model R&D, we construct corresponding indicators for non-key core technologies R&D and utility model R&D based on the two indicators of key core technologies R&D. Non-key core technologies R&D includes invention patents that are not key core technologies, while utility model R&D includes utility model patents. The calculation methods for these indicators are the same as described above.

#### 3.3.2. Independent variable.

The independent variable is the science and technology talent policies. This study employs a multi-period DID model, we constructed a dummy variable TP as follows:


TP=treat×time (3)

where treat denotes the group implementing the science and technology talent policies, and time denotes the period during which the policy was implemented. The enterprises receiving support from the science and technology talent policies are assigned a value of 1 for treat, while those that did not receive support are assigned a value of 0. For the years in which an enterprise received policy support, and for subsequent years, time is assigned a value of 1, with all other years assigned a value of 0.

Furthermore, to further examine the heterogeneous effects of science and technology talent policies on key core technologies R&D, we analyze the support intensity and types of science and technology talent policies. Regarding the support intensity of the policies, although the specific content of the talent policies cannot be directly quantified, each policy has an associated funding amount. We analyzed the sample data and, referring to the support standards of science and technology talent policies across different government levels and regions, categorized the policy support into three levels based on the amount of funding. Policies with funding above 1 million yuan are classified as high-level, policies with funding between 300000 and 1 million yuan are classified as medium-level, and policies with funding below 300000 yuan are classified as low-level. The proportions of high-level, medium-level, and low-level science and technology talent policies in the sample are approximately 17.14%, 27.11%, and 55.75%, respectively. Regarding the types of science and technology talent policies, we categorized them based on the policy background, and analyzed four types of policies: talent subsidies, innovation incentives, talent introduction, and high-level talent innovation support.

Additionally, in the robustness check for substituting the independent variable, the support funding amount for science and technology talent policies is used as a quantifiable indicator of the policy, and a panel regression model is constructed. The ordinary least squares (OLS) method is employed for the regression.

#### 3.3.3. Control variables.

In order to reduce the estimation bias caused by omitted variables, this study adds a series of control variables aimed at controlling other factors that may affect the key core technologies R&D of enterprises. Based on the criteria of relevant theoretical research and data availability, the factors that have been widely verified in the relevant literature as likely to affect enterprises’ innovation capability and technology R&D are selected. Specifically, four categories are included: basic enterprise characteristics, governance structure, financial status and market performance.

(1) Basic enterprise characteristics

The stage of development of an enterprise has a positive impact on its innovative capacity [45]. Enterprises that have been established for a longer period of time may have accumulated more technology and experience, which is conducive to innovation investment, but may also be less motivated to innovate due to organizational inertia [46]. Therefore, this study controls for the following variables:

The enterprise’s years since establishment (CV1): measures the history of the enterprise.

The years since the enterprise went public (CV2): reflects the time when the enterprise enters the capital market, which affects its financing ability and innovation investment.

(2) Governance structure

The equity structure of an enterprise affects the performance of technological innovation [47]. Enterprises’ ownership structure affects innovation by influencing the allocation of innovation resources, and it has been found that enterprises with a more concentrated ownership structure can quickly reach the optimal level of innovation performance [48]. Accordingly, this study controls for the following variables:

The ownership ratio of the top ten shareholders (CV3): measures the impact of equity concentration on enterprises’ decision-making.

The ownership ratio of the management (CV4): reflects the degree of interest binding of managers, which affects innovation incentives.

(3) Financial status

An enterprise’s financial resources and asset structure determine its ability to invest in R&D [49]. Adequate cash flow may provide more sufficient funds for enterprise innovation, while a high proportion of fixed assets may limit the flexible use of funds. Therefore, this study controls for the following variables:

The number of employees (CV5): measures the enterprise’s human capital investment, which is related to the allocation of R&D resources.
The enterprise size (SA value) (CV6): controls for the impact of enterprise size on innovation.
Total operating income (CV7): reflects the market competitiveness and innovation investment capacity of the enterprise.
Cash ratio (CV8): measures the liquidity of the enterprise.
Quick ratio (CV9): measures the short-term solvency of the enterprise and reflects the financial health.
Intangible assets ratio (CV10): a higher proportion of intangible assets may imply stronger intellectual property protection and innovation capability.
Fixed assets ratio (CV11): excessive fixed assets may limit an enterprise’s flexibility to invest in innovation.
Current asset ratio (CV12): Measures whether an enterprise’s asset structure supports innovation activities.

(4) Market Performance

The market valuation of an enterprise reflects external investors’ expectations of its future growth and also affects the an enterprise’s innovation strategy, Tobin’s Q is often used to measure the market value and innovation potential of an enterprise [50]. Therefore, this study controls for the following variables:

Tobin’s Q ratio (CV13): The Tobin’s Q ratio is calculated as follows.


$$Tobin^{\prime }s\;Q\text{=}\frac{market\;value\;of\;equity\;+\;market\;value\;of\;net\;debt}{total\;assets\;at\;year-end}$$ (4)

The data for the above variables are obtained from annual and financial reports of listed enterprises to ensure the reliability and comparability of the data. Controlling these variables helps to reduce other factors that may affect the key core technologies R&D of enterprises, so as to more accurately identify the role of science and technology talent policies.

### 3.4. Sample and data sources

#### 3.4.1. Sample selection and treatment group division.

(1) Sample Selection

The sample selection is primarily based on two criteria: First, a sufficient number of sample enterprises must be ensured, as there are relatively few enterprises that receive support from science and technology talent policies and possess key core technologies patents. Moreover, as the level of science and technology talent policies increases, the number of policies continues to decrease, so it is essential to guarantee a sufficiently large sample size. Second, the sample enterprises must have a long continuous time series, as science and technology talent policies and key core patents only appear in certain years. The probability of encountering both increases as the number of continuous years in the sample increases, which helps to improve the identification of the policy effect. In addition, the consecutive time horizon can fully take into account the time effect of policy implementation and the potential differences between enterprises in different years. Based on the “National Standards of the People’s Republic of China (GB/T 4754-2017)”, we select publicly listed enterprises in the subsectors of the manufacturing industry and the information transmission, software, and information technology services industry as the sample. These industries are the primary sources of key core patents and are also the main areas of technological competition worldwide, making the study of these industries of significant practical relevance. In addition, we select the period from 2011 to 2023 as the sample years, and based on this, we construct the sample data. Descriptive statistics of the data are presented in Table 1.

**Table 1:** Descriptive statistics of variables.

Variable	Observation	Mean	Standard Deviation	Minimum	Maximum
KCT1	17719	1.272	9.226	0.000	403.000
KCT2	17719	0.147	0.356	0.000	5.872
TP	17719	0.393	0.488	0.000	1.000
CV1	17719	0.043	0.037	0.000	0.517
CV2	17719	12.760	7.153	0.000	33.000
CV3	17719	53.488	15.116	0.000	95.499
CV4	17719	10.341	17.087	0.000	89.725
CV5	17719	5996.782	15391.450	0.000	703504.000
CV6	17719	22.258	1.404	0.000	27.640
CV7	17719	9.24E + 09	3.11E + 10	0.000	9.02E + 11
CV8	17719	0.932	2.773	-0.013	167.544
CV9	17719	2.052	3.759	0.000	179.578
CV10	17719	0.045	0.042	0.000	0.677
CV11	17719	0.223	0.146	0.000	0.872
CV12	17719	0.567	0.175	0.000	1.000
CV13	17719	2.126	2.404	0.000	122.190

(2) Treatment Group Division

On the basis of sample selection, we screened out the enterprises that have received support from science and technology talent policies based on the data of the sample enterprises downloaded from the CSMAR (China Stock Market & Accounting Research) database. In this study, we screened out the funding projects related to the science and technology talent policies from various government funding titles, and the specific screening keywords include “talent, doctor, academician, leading, high-level, expert, outstanding young scholars, young scholars, professor, overseas, and team”. During the screening process, two researchers independently check the screening results to ensure that each funding information is related to the science and technology talent policies. Based on the screening results, we labeled the enterprises that had received policy support as the treatment group (treat = 1) and those that had not received policy support as the control group (treat = 0). This division ensures that the difference between the treatment group and the control group before the implementation of the science and technology talent policies is minimized, thus enhancing the validity of the multi-period DID model analysis. To further ensure comparability between the treatment and control groups, we also used propensity score matching to match the samples and control for selection bias.

#### 3.4.2. Data sources.

Patent data were sourced from the Patyee database, which covers nearly 180 million patent records from over 171 countries, regions, and organizations worldwide. Using this database, we filtered all patent information for each sample enterprise in the selected years, and from this, calculated the relevant indicators.

The data on science and technology talent policies, control variables, and robustness tests were obtained from the CSMAR (China Stock Market & Accounting Research) database. This database is designed specifically for academic research and quantitative investment analysis, drawing on professional standards from internationally recognized databases such as CRSP and COMPUSTAT while also incorporating the specific circumstances of China. It provides a comprehensive and authoritative set of financial and economic data, including over 4,000 tables and more than 50,000 fields. We extracted the necessary fields for the listed enterprises from this database to calculate the relevant indicators.

## 4. Empirical analysis

### 4.1. Propensity score matching

In this study, enterprises that receive talent policies and those that do not may exhibit significant differences across multiple dimensions. These differences are not only reflected in observable variables but may also be influenced by unobservable factors. Conducting regression analysis in the presence of selection bias could lead to the underestimation or overestimation of the policy effect, thus affecting the accuracy of causal inference. To address this issue, this study employs the propensity score matching (PSM) method to handle sample selection bias and the heterogeneity between the treatment group and the control group caused by unobservable factors.

Specifically, propensity score matching first calculates the propensity score for each enterprise, which represents the probability of the enterprise being assigned to the treatment group. This calculation helps address the issue of selection bias. In this study, 13 control variables are selected to fully reflect the basic characteristics of enterprises and compute their propensity scores. Then, based on the propensity scores, enterprises with similar scores in the treatment and control groups are matched, thus controlling for potential confounding factors and ensuring comparability in key characteristics after matching. Through this matching process, this study effectively reduces systematic differences between the samples, providing a more reliable basis for estimating the effect of talent policies.

The results of the propensity score matching calculations are shown in Table 2, where the bias between the treatment group and the control group in all variables has been significantly reduced. According to the matched mean values and t-test results, the bias (% Bias) for all variables has been reduced to a low level, and the t-values and p-values indicate that the differences between the treatment and control groups are no longer significant. Although the p-value for the CV3 variable is slightly above 0.05 and approaches the level of significance, it has not yet reached a significant threshold, suggesting that the bias for this variable is within an acceptable range. Overall, the propensity score matching has effectively reduced potential selection bias, enhancing the comparability between treatment and control groups in key characteristics. Subsequent analyses will be based on the results of propensity score matching to ensure that the regression results are more robust and reliable.

**Table 2:** Results of propensity score matching.

Variables	Mean			T-test	
	Treated mean	Control mean	% Bias	T-value	P-value
CV1	2.941	2.937	1.300	0.950	0.341
CV2	2.364	2.366	-0.300	-0.190	0.852
CV3	3.948	3.939	2.600	1.910	0.056
CV4	1.456	1.427	1.900	1.340	0.182
CV5	7.832	7.842	-0.700	-0.560	0.578
CV6	3.142	3.142	0.400	0.370	0.711
CV7	21.557	21.535	1.400	1.080	0.280
CV8	0.480	0.473	1.400	0.970	0.330
CV9	0.950	0.938	2.300	1.630	0.104
CV10	0.042	0.042	0.500	0.430	0.665
CV11	0.190	0.188	1.300	0.960	0.338
CV12	0.449	0.448	1.000	0.740	0.460
CV13	1.044	1.040	1.100	0.830	0.406

### 4.2. Baseline regression analysis

Columns (1) and (2) of Table 3 report the regression results of science and technology talent policies (TP) on the two indicators of key core technologies R&D (KCT). The estimated coefficients for TP range from 0.013 to 0.027, and all coefficients are statistically significant, indicating that TP has a significant positive effect on KCT. To further understand this impact, we regress TP on non-key core invention patents (NKP) and utility model patents (UM), using the same quantification methods as for the two KCT indicators. In columns (3) through (6), the estimated coefficients for TP are positive and statistically significant at the 1% level, suggesting that TP also promotes NKP and UM. By calculating the ratios of the estimated coefficients for the two KCT indicators to those for NKP, we obtain 0.108 and 0.333; similarly, the ratios for KCT and UM are 0.365 and 0.684. We find that the positive effect of TP on KCT is the smallest, the effect on NKP is the largest, and the effect on UM is intermediate. This may be because key core technologies R&D involves higher technical barriers and complexity, and under the constraints of R&D assessments, talent is more inclined to focus on general technologies with relatively lower difficulty. This suggests that talent policies can promote key core technologies R&D, although this effect is weaker than for non-key core technologies.

**Table 3:** Baseline regression results.

Variables	Key core technologies R&D		Non-key core technologies R&D		Utility model R&D	
	(1)	(2)	(3)	(4)	(5)	(6)
	KCT1	KCT2	NKP1	NKP2	UM1	UM2
TP	0.027^**^	0.013^***^	0.249^***^	0.039^***^	0.074^***^	0.019^***^
t-Value	2.390	3.090	9.830	7.460	3.320	4.000
CV	yes	yes	yes	yes	yes	yes
year	yes	yes	yes	yes	yes	yes
industry	yes	yes	yes	yes	yes	yes
C	-0.816^***^	-0.238^***^	-1.591^***^	0.512^***^	0.354	0.205^***^
t-Value	-2.590	-2.680	-2.600	9.120	1.110	3.230
F	44.910	61.430	43.350	210.010	85.820	47.160
Prob	0.000	0.000	0.000	0.000	0.000	0.000
Adj R^2^	0.223	0.193	0.136	0.241	0.212	0.129

* ,

** , and

*** indicate significance at the 10%, 5%, and 1% levels, respectively.

### 4.3. Regression analysis of science and technology talent policies with different levels of support

The regression results of science and technology talent policies with different levels of support are presented in Table 4. Due to space limitations, only the estimated coefficients, t-statistics, and significance levels of the independent variables are shown. The estimated models, control variables, fixed effects, and sample size are consistent with those in the baseline regression. It can be observed that the estimated coefficient of the advanced science and technology talent policies (ATP) for KCT ranges from 0.034 to 0.113 and is statistically significant at the 1% level, indicating that ATP has a significant positive effect on KCT. The estimated coefficient of the intermediate science and technology talent policies (ITP) for KCT ranges from 0.026 to 0.049 and is also significant at the 1% level, indicating that ITP also has a significant positive impact on KCT. However, the estimated coefficient of the low science and technology talent policies (LTP) for KCT is not significant and is relatively small. By calculating the ratio of the estimated coefficients of ATP to those of ITP and LTP for the two indicators of KCT, we obtain the values 2.306, 1.308, 12.556, and 5.667. This suggests that ATP has the greatest impact on KCT, followed by ITP, with LTP having the smallest impact. This indicates that as the support intensity of science and technology talent policies increases, their impact on key core technologies R&D also grows. This conclusion is also confirmed by the regression results of TP on NKP1, but cannot be confirmed for TP on NKP2. In the regression results of TP on UM, although ATP’s impact remains the greatest, the estimated coefficient of ITP for UM1 is slightly smaller than that of LTP.

**Table 4:** Regression results of talent policies with different levels of support.

Variables	Key core technologies R&D		Non-key core technologies R&D		Utility model R&D	
	(1)	(2)	(3)	(4)	(5)	(6)
	KCT1	KCT2	NKP1	NKP2	UM1	UM2
ATP	0.113^***^	0.034^***^	0.443^***^	0.029^***^	0.170^***^	0.028^***^
t-Value	5.220	4.700	10.440	4.170	4.590	3.600
ITP	0.049^***^	0.026^***^	0.274^***^	0.030^***^	0.030^***^	0.021^***^
t-Value	3.210	4.650	8.490	5.300	1.060	3.400
LTP	0.009	0.006	0.153^***^	0.031^***^	0.075^***^	0.015^***^
t-Value	0.820	1.410	5.920	5.820	3.290	2.990

* ,

** , and

*** indicate significance at the 10%, 5%, and 1% levels, respectively.

Further comparison of the estimated coefficients for KCT, NKP, and UM under similar science and technology talent policies shows that ATP has the largest estimated coefficient for NKP1, the second largest for UM1, and the smallest for KCT1; for KCT2, ATP has the largest estimated coefficient, followed by NKP2, with UM2 having the smallest. ITP has the largest estimated coefficient for NKP, the second largest for KCT, and the smallest for UM. LTP has the largest estimated coefficient for NKP, the second largest for UM, and the smallest for KCT. It can be observed that, except for ATP’s estimated coefficient for KCT2, the promoting effect of science and technology talent policies at all three support levels on KCT is not the largest. This suggests that, compared to non-key core technologies R&D and utility model R&D, the promoting effect of science and technology talent policies at varying levels of support on key core technologies R&D is relatively smaller. This conclusion is consistent with the regression results in Table 3.

### 4.4. Regression analysis of different types of science and technology talent policies

The regression results for different types of science and technology talent policies are shown in Table 5. Due to space limitations, only the estimated coefficients, t-statistics, and significance levels of the independent variables are presented. The estimated models, control variables, fixed effects, and sample size are consistent with those in the baseline regression. This section conducts regression analyses for four types of talent policies: talent subsidies (TP1), innovation incentives (TP2), talent introduction (TP3), and high-level talent innovation support (TP4), based on the policy background described earlier. It can be observed that all four types of talent policies have a significant positive impact on KCT. For KCT1, among the four talent policies, TP1 has the strongest positive impact, with a coefficient of 0.079, followed by TP2, TP3, and TP4, with TP4’s impact being noticeably smaller than the other three policies. For KCT2, TP2’s positive impact is significantly higher than the other three, while TP4’s positive impact remains the smallest. Considering both KCT1 and KCT2, TP4 has the smallest positive effect. This may be because TP4 focuses on long-term support for high-level talents, whose effects are usually more indirect and depend on the synergy with other resources. The introduction, cultivation, and innovation outcomes of high-level talents often take a long time to materialize, and their contributions are more apparent in strategic decision-making and guiding technological directions rather than directly driving specific technological research and development.

**Table 5:** Regression results of different types of talent policies.

Variables	Key core technologies R&D		Non-key core technologies R&D		Utility model R&D	
	(1)	(2)	(3)	(4)	(5)	(6)
	KCT1	KCT2	NKP1	NKP2	UM1	UM2
TP1	0.079^***^	0.023^***^	0.337^***^	0.026^***^	0.225^***^	0.025^***^
t-Value	5.140	4.050	9.300	3.140	7.060	3.520
TP2	0.076^***^	0.062^***^	0.464^***^	0.046^***^	0.362^***^	0.028^***^
t-Value	4.160	6.170	10.850	4.630	9.650	3.340
TP3	0.071^***^	0.028^***^	0.276^***^	0.023^**^	0.219^***^	0.047^***^
t-Value	3.730	3.960	4.150	2.210	5.580	5.410
TP4	0.043^**^	0.021^***^	0.330^***^	0.052^***^	0.258^***^	0.027^***^
t-Value	1.990	3.430	8.300	5.720	7.400	3.430

* ,

** , and

*** indicate significance at the 10%, 5%, and 1% levels, respectively.

Additionally, further comparative analysis of the four types of talent policies’ impact on NKP and UM shows that all four talent policies have significant positive impact on both NKP and UM. Overall, the four talent policies have the strongest effect on NKP, followed by UM, with the smallest effect on KCT. When considering both NKP1 and NKP2, TP3 has the smallest positive impact, while for UM1 and UM2, the positive impact of different types of talent policies varies greatly. In general, the four talent policies have a significant positive effect on technological research and development, suggesting that although these policies have different focal points, they each play to their strengths, supporting technological research and development from different angles. This provides strong empirical support for policymakers. Moreover, this conclusion is consistent with the baseline regression results and the results for the intensity of talent policies support, further confirming the robustness of the above regression outcomes.

Whether there are cumulative or offsetting effects between different types of science and technology talent policies needs to be further analyzed by adding the interaction terms between the four types of science and technology talent policies in model (1), and the results are shown in Table 6. First, analyzing columns (1) to (2), except for the regression result of TP4 on KCT1 which is not significant, the other regression results are all significant and positive, which indicates that there is a facilitating effect of the four types of talent policies on KCT. After that, columns (3) to (14) are analyzed, and it is found that except for the coefficient of TP1 × TP2 and the coefficient of TP1 × TP4 which is not significant, the coefficients of the interaction terms are all significantly negative, and the regression coefficients of each policy are all significantly positive, which indicates that there is no significant interaction effect between TP1 and TP2 and TP4, and that there is no significant interaction effect between the other. There is an offsetting effect between the two talent policies. In addition, we also find that the regression coefficients of the four types of talent policies increase after the introduction of the interaction term compared with the model without the interaction term. The main reason for this phenomenon is that the estimation of the interaction term adjusts for the independent effects of individual policies. Specifically, the negative regression coefficients of the interaction terms indicate that there may be offsetting effects for certain policy combinations, i.e., when two types of policies are implemented simultaneously, their combined effects are smaller than the simple sum of their individual effects. This means that the regression coefficients of individual policies may partially absorb these interactions when the interaction term is not introduced, whereas after the addition of the interaction term, these effects are disaggregated, resulting in an increase in the coefficients of the direct impacts of individual policies. Therefore, in understanding the role of various types of talent policies, the direct effects of individual policies as well as the interactions between policies should be taken into account.

**Table 6:** Regression results on the interaction between talent policies.

Variables	(1)	(2)	(3)	(4)	(5)	(6)	(7)	(8)	(9)	(10)	(11)	(12)	(13)	(14)
	KCT1	KCT2	KCT1	KCT2	KCT1	KCT2	KCT1	KCT2	KCT1	KCT2	KCT1	KCT2	KCT1	KCT2
TP1	0.070^***^	0.018^***^	0.074^***^	0.022^***^	0.104^***^	0.031^***^	0.079^***^	0.024^***^						
t-Value	4.490	3.190	4.480	3.630	6.220	5.060	4.740	3.850						
TP2	0.069^***^	0.039^***^	0.068^***^	0.042^***^					0.091^***^	0.047^***^	0.091^***^	0.048^***^		
t-Value	3.750	5.770	3.310	5.560					4.770	6.700	4.550	6.480		
TP3	0.061^***^	0.024^***^			0.114^***^	0.043^***^			0.088^***^	0.034^***^			0.100^***^	0.036^***^
t-Value	3.160	3.420			5.160	5.290			4.380	4.610			4.810	4.660
TP4	0.024	0.017^***^					0.031^*^	0.022^***^			0.044^**^	0.026^***^	0.054^***^	0.027^***^
t-Value	1.420	2.780					1.660	3.210			2.420	3.800	2.980	4.050
TP1×TP2			0.013	-0.010										
t-Value			0.280	-0.570										
TP1×TP3					-0.209^***^	-0.071^***^								
t-Value					-4.750	-4.410								
TP1×TP4							-0.004	-0.009						
t-Value							-0.080	-0.570						
TP2×TP3									-0.177^***^	-0.066^***^				
t-Value									-2.760	-2.810				
TP2×TP4											-0.100^**^	-0.044^**^		
t-Value											-2.070	-2.460		
TP3×TP4													-0.190^***^	-0.056^***^
t-Value													-3.790	-3.050
CV	yes	yes	yes	yes	yes	yes	yes	yes	yes	yes	yes	yes	yes	yes
year	yes	yes	yes	yes	yes	yes	yes	yes	yes	yes	yes	yes	yes	yes
industry	yes	yes	yes	yes	yes	yes	yes	yes	yes	yes	yes	yes	yes	yes
C	-0.591^***^	-0.190^**^	-0.585^***^	-0.185^**^	-0.587^***^	-0.185^**^	-0.589^**^	-0.188^**^	-0.581^**^	-0.183^**^	-0.595^**^	-0.190^**^	-0.580^**^	-0.186^**^
t-Value	-2.530	-2.200	-2.500	-2.150	-2.510	-2.150	-2.510	-2.180	-2.480	-2.130	-2.540	-2.210	-2.480	-2.150
F	16.610	13.980	16.610	13.950	16.680	13.940	16.570	13.860	16.600	13.990	16.550	13.960	16.580	13.890
Prob	0.000	0.000	0.000	0.000	0.000	0.000	0.000	0.000	0.000	0.000	0.000	0.000	0.000	0.000
Adj R^2^	0.242	0.210	0.241	0.209	0.242	0.209	0.241	0.208	0.241	0.209	0.241	0.209	0.241	0.208

* ,

** , and

*** indicate significance at the 10%, 5%, and 1% levels, respectively.

### 4.5. Parallel trend test and sensitivity analysis

**Fig 2:**
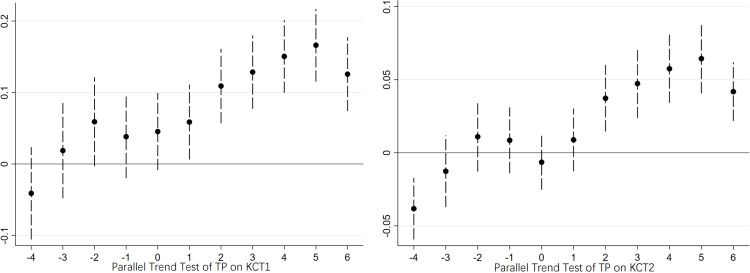
Parallel trend test.

This study conducts a parallel trend test to verify the trend differences between the treatment group and the control group before and after the policy implementation. Specifically, a relative time framework is adopted, where the policy implementation period is defined as 0, the periods before implementation are those before 0, and the periods after implementation are those after 0. The parallel trend assumption requires that, prior to the policy implementation, the economic trends of the treatment and control groups should be parallel. Therefore, if, before the policy implementation, the regression coefficients for the treatment and control groups are close to 0 and their confidence intervals contain 0, it can be concluded that there is no significant difference in the trends between the two groups, thus passing the parallel trend test. Based on this testing method, the results are shown in Fig 2. In Fig 2, the horizontal axis represents the relative periods based on the policy shock, and the vertical axis represents the effect of the science and technology talent policies. The black dots represent the regression coefficients for the effect of the science and technology talent policies on key core technologies R&D, and the dashed lines represent the 95% confidence intervals.

From the figure, it can be observed that, before the policy implementation, the regression coefficients for the treatment and control groups fluctuate around 0, and the 95% confidence intervals mostly include 0, indicating that there is no significant difference in trends between the two groups before the implementation, thus validating the parallel trend assumption. Specifically, between periods -4 and -1, except for period -4, the regression coefficients for the other periods do not significantly deviate from 0, further supporting the validity of the parallel trend assumption. Regarding the dynamic effects after the policy implementation, the regression analysis indicates that, in the policy implementation period and the first period after implementation, the regression coefficients are not significant, suggesting that the policy’s impact does not immediately manifest. Starting from the second period, the regression coefficients are significantly positive, indicating that the policy begins to have a positive impact on key core technologies R&D. This effect gradually weakens in periods 5 and 6, showing a typical lag effect. This result suggests that the science and technology talent policies have a sustained positive effect on R&D investment in the first five years after its implementation, after which the effect starts to gradually diminish. Overall, the parallel trend test results pass, indicating that there is no significant difference in trends between the treatment and control groups before the policy implementation.

**Fig 3:**
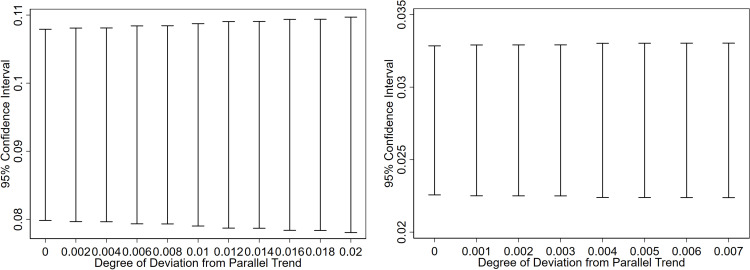
Sensitivity analysis of parallel trend with relative deviation limits.

**Fig 4:**
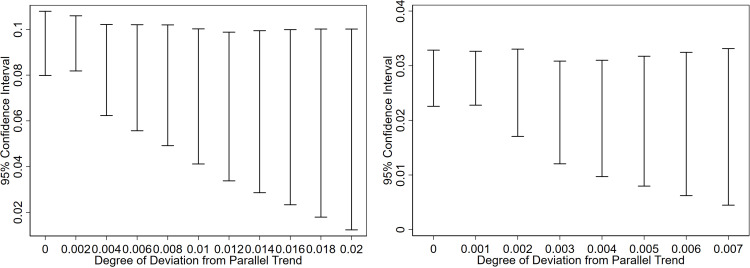
Sensitivity analysis of parallel trend under smoothing restrictions.

It has been found that the traditional ex ante parallel trend test remains statistically insufficiently valid and may lead to estimation bias [51]. Therefore, to further test the robustness of the effects of science and technology talent policies, we employ parallel trend sensitivity analysis to assess the robustness of the policy effects in the presence of potential parallel trend deviations. Specifically, the method sets the maximum degree of deviation from the parallel trend and examines whether the estimated coefficients of the treatment effects remain significant at that degree of deviation. If the confidence interval of the estimated coefficients still does not contain zero even at the maximum degree of deviation, it indicates that the policy effect is robust to parallel trend deviations. Based on the existing literature, we adopt a stricter criterion by setting the maximum deviation degree as two times the standard error of the estimated coefficients at the post-treatment point, and conduct a sensitivity analysis for the estimated coefficients in the first period after the policy shock. Specifically, for the indicators of key core technologies R&D, KCT1 and KCT2, the standard errors of the point estimates for the first year after the science and technology talent policies shock are about 0.01 and 0.0035, respectively, and thus we set the maximum degree of deviation to 0.02 and 0.007, respectively, to test the robustness of the policy effects in the presence of parallel trend deviations. The results of the analysis are shown in Figs 3 and 4, where Fig 3 demonstrates the results of the parallel trend sensitivity analysis under the relative deviation degree restriction and Fig 4 presents the results of the parallel trend sensitivity analysis under the smoothing restriction. Among them, the left figure corresponds to KCT1, and the right figure corresponds to KCT2. From the results, the confidence intervals of the estimated coefficients do not contain 0 under different deviation degree settings, indicating that the promotion effect of science and technology talent policies on key core technologies R&D has strong robustness.

## 5. Robustness checks

### 5.1. Placebo test

**Fig 5:**
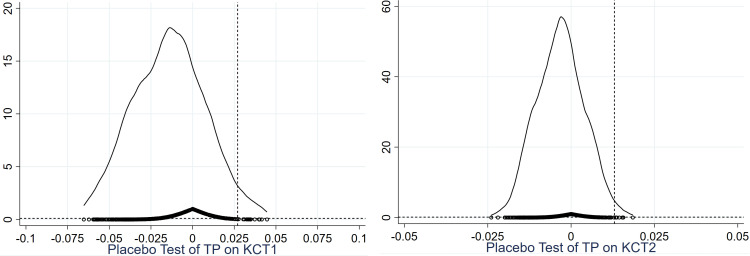
Placebo test.

To verify that the impact of science and technology talent policies on key core technologies R&D is genuine and not driven by unobservable factors, a placebo test is conducted. The basic idea of the test is to randomly select a subset of samples from all the samples to form a treatment group, then perform a regression to obtain the estimated coefficient for the science and technology talent policies. This process is repeated 500 times, and the results are plotted as a kernel density plot, as shown in Fig 5. The dashed line in the figure represents the true estimated coefficient for the science and technology talent policies on key core technologies R&D, as reported in Table 3. It can be observed that the true estimated coefficients for both indicators are far from the mean of the kernel density distribution, indicating that the science and technology talent policies indeed have a positive impact on key core technologies R&D, and that this effect is not due to unobservable factors.

### 5.2. Regression analysis of the different number of patents

In the baseline regression analysis, the impact of science and technology talent policies on the number of citations of the top 5% of invention patents was examined. However, as mentioned in the research design, there is no specific standard for measuring key core patents according to existing studies. Therefore, in addition to the top 5% criterion, we will expand the range of patent analysis to include patents ranked in the top 1%, top 10%, top 15%, top 20%, and top 25% based on the number of citations. Additionally, patents exhibit a time lag; for instance, there is a lag between the time when enterprises start R&D after receiving talent policies support, the submission of patent applications, the granting of patents, and their citation. This suggests that failing to account for time lags in the regression results could lead to bias. However, due to differences in the technological fields of patents and their technological complexity, it is difficult to identify a specific universally applicable lag period. Therefore, this study conducts regression analysis using lags ranging from 1 to 5 periods. The regression results are presented in Table 7.

**Table 7:** Regression results of the different number of patents.

Independent variables	(1)	(2)	(3)	(4)	(5)
	Lag 1 period	Lag 2 period	Lag 3 period	Lag 4 period	Lag 5 period
P1	0.00078	0.00048	0.00095	0.00008	-0.00117
t-Value	0.51	0.29	0.51	0.04	-0.49
P5	0.021^**^	0.016	0.015	0.017	0.018
t-Value	1.99	1.38	1.19	1.17	1.08
P10	0.033^**^	0.030^**^	0.025^*^	0.026	0.029^*^
t-Value	2.43	2.09	1.60	1.54	1.60
P15	0.059^***^	0.058^***^	0.056^***^	0.053^***^	0.054^***^
t-Value	3.81	3.55	3.24	2.81	2.64
P20	0.078^***^	0.072^***^	0.069^***^	0.063^***^	0.058^***^
t-Value	4.68	4.07	3.64	3.09	2.64
P25	0.099^***^	0.094^***^	0.088^***^	0.079^***^	0.071^***^
t-Value	5.57	4.98	4.39	3.63	3.02

* ,

** , and

*** indicate significance at the 10%, 5%, and 1% levels, respectively.

Due to the large volume of data from the estimates, it is not feasible to present all the results. Therefore, only the regression coefficients, significance levels, and t-values for the effect of talent policies on the citation counts of patents ranked in the top 1% (P1), top 5% (P5), top 10% (P10), top 15% (P15), top 20% (P20), and top 25% (P25) with lags of 1–5 periods are presented. From the estimation results, it can be observed that the estimated coefficients for P1 are very small and insignificant. This is likely due to the limited number of patents in the top 1% category, leading to insignificant regression results. For P5, the estimated coefficient at lag 1 is 0.021 and is statistically significant at the 5% level. From lag 2 to lag 5, the estimated coefficients are not significant, although they remain positive, indicating that talent policies have a short-term lagged positive impact on the number of patents ranked in the top 5%. Additionally, for P10, P15, P20, and P25, the estimated coefficients are positive and significant, with only the estimated coefficient for P10 at lag 4 being insignificant. Overall, talent policies have a positive impact on high-citation patents. From the same lag period, as the number of highly cited patents increases, the estimated coefficients for the number of patents also grow larger, suggesting that the effect of talent policies becomes more pronounced with an increase in the number of patents. Regarding the ordering of the lag periods, the estimated coefficients for the number of patents generally decrease as the lag period increases. Comparing the estimated coefficients for lag 5 with lag 1, it can be found that for P5 to P25, the decline in the effect of talent policies ranges from 8.475% to 28.283%, indicating that the impact of talent policies on patent numbers exhibits a time lag and that the effect weakens over time.

### 5.3. Solution to the heterogeneous treatment timing problem

In the process of studying the impact of science and technology talent policies on key core technologies R&D, the variation in the timing of talent policies implementation across different enterprises in the sample leads to a heterogeneous treatment timing problem. This issue can result in dynamic changes between the treatment and control groups, thereby affecting the accuracy and robustness of the estimated results. To address this potential bias, this study adopts methods from the relevant literature to correct for the estimation bias induced by heterogeneous treatment timing, ensuring the robustness and interpretability of the empirical results.

Due to the significant heterogeneity in treatment effects arising from the different timing of talent policies implementation across enterprises in the sample, the method proposed by Callaway (2021) is more suitable than other methods (such as the methods of Sun and Abraham (2021) and stacked regression) [52]. This method allows for the precise estimation of specific effects for each period and treatment group, capturing the time-varying nature of policy effects while flexibly choosing the control group (which can include untreated enterprises or enterprises that receive treatment later). This approach effectively avoids estimation bias resulting from inappropriate control group selection. Furthermore, Callaway’s method allows for the inclusion of covariates to control for enterprise characteristics, significantly reducing estimation errors caused by selection bias and improving the robustness and interpretability of the results. In contrast, Sun and Abraham’s method provides weaker support for covariate control and imposes strict conditions on the control group selection, while the stacked regression method, although simple, has limited flexibility in handling long-term dynamic effects and time-lagged effects, making it less suitable for samples with substantial differences in policy implementation timing across multiple years. Callaway’s method, through various robust estimation techniques (such as inverse probability weighting and double-robust methods), further enhances the reliability of the estimates, particularly in scenarios where policy effects exhibit dynamic trends over time. Therefore, this study selects Callaway’s method to accurately capture the dynamic characteristics of policy effects and ensure the robustness of the empirical results.

At the same time, we also consider the issue of lagged effects in the impact of talent policies on the number of patents and the number of citations of patents. We conduct regressions using data on KCT1 and KCT2, as well as their lagged values from 1 to 5 periods, with the results presented in Table 8. In the estimation method, we adopt a dynamic reweighted multi-period DID method. This method adjusts the weights for each period dynamically, providing more robust estimates when facing heterogeneous treatment effects. We estimate the treatment effects using a simple aggregation method, directly averaging the effects across all treatment periods. After controlling for covariates, years, and industries, the average treatment effect of the talent policies (TP-ATT) is significant in columns (1) and (2), indicating that the talent policies still have a positive impact on KCT1, as well as on its lagged value by one period. The TP-ATT is also significant in columns (7) to (9), demonstrating that the talent policies continue to have a positive impact on KCT2, including its lagged values by one and two periods. This suggests that the talent policies have a significant promoting effect on key core technologies R&D.

**Table 8:** Regression results of the Callaway method.

Variables	KCT1						KCT2					
	(1)	(2)	(3)	(4)	(5)	(6)	(7)	(8)	(9)	(10)	(11)	(12)
	Current period	Lag 1 period	Lag 2 period	Lag 3 period	Lag 4 period	Lag 5 period	Current period	Lag 1 period	Lag 2 period	Lag 3 period	Lag 4 period	Lag 5 period
TP-ATT	0.049^**^	0.044^**^	0.031	0.033	0.002	-0.033	0.016^*^	0.021^**^	0.017^*^	0.011	-0.005	-0.015
Std. Err.	0.021	0.022	0.023	0.024	0.026	0.029	0.008	0.009	0.009	0.010	0.011	0.012
Z-Value	2.340	1.990	1.370	1.360	0.090	-1.110	1.910	2.290	1.850	1.100	-0.470	-1.250
CV	yes	yes	yes	yes	yes	yes	yes	yes	yes	yes	yes	yes
year	yes	yes	yes	yes	yes	yes	yes	yes	yes	yes	yes	yes
industry	yes	yes	yes	yes	yes	yes	yes	yes	yes	yes	yes	yes

* ,

** , and

*** indicate significance at the 10%, 5%, and 1% levels, respectively.

### 5.4. Quantile regression analysis

The analysis above supports the hypothesis that science and technology talent policies promote key core technologies R&D. However, in the robustness checks, the regression analysis was conducted only on the number of patents, and in the baseline regression, we focused solely on the number of citations of the top 5% of patents, lacking a deeper discussion of this metric. Additionally, the number of citations of patents exhibit a more significant lag than the patent grant time. Research has shown that patent citations can trace back many years: 50% of citations are directed towards patents granted at least 10 years prior, 25% to patents granted at least 20 years earlier, and 5% to patents granted at least 50 years earlier [53]. Therefore, the analysis of patent citations requires accounting for lag. Since the sample period of this study is from 2011 to 2023, and the patent citation data collected extends only through the end of 2024, with citations after that year being unknown, the fixed effects method proposed by Bronwyn H Hall is employed to adjust for the number of citations of patents. This allows for comparable the number of citations across time and technological fields, with the adjustment method detailed in the research design section. Furthermore, as there is a significant time lag between an enterprise receiving talent policies support, conducting R&D, and having patents cited, we conduct regression analyses using lagged data from 1 to 8 periods. This is because the data ends in 2023, and using lagged data up to the 8th period ensures that at least five years of continuous data are available for regression, thus enhancing the robustness of the results.

In contrast to the previous section, this part employs quantile regression to analyze the effects at the 99th percentile, 95th percentile, 90th percentile, 85th percentile, 80th percentile, and 75th percentile. The number of citations of patents are well-suited for quantile regression because they often exhibit significant right-skewed distributions, with most patents being cited infrequently and only a small number of patents receiving a very high number of citations. This heterogeneity makes quantile regression suitable for revealing the differential effects of science and technology talent policies at various quantiles. Therefore, quantile regression better captures the diversity and nonlinear characteristics of its impacts when applied to the number of citations.

The regression results are presented in Table 9, where PC represents the number of citations of patents, and the numbers following it refer to different percentiles. Since the regression results after the 6th lag are not significant, they are not shown. It can be observed that, from the current period to the 6th lag, at the 99th percentile, the estimated coefficient for the number of citations of patents is small and insignificant, which may be due to the small sample size. Between the 90th percentile and the 75th percentile, all estimated coefficients are significantly positive from the current period to the 4th lag. The estimates for the 5th lag are insignificant at the 90th and 75th percentiles, and estimates for the 6th lag and beyond are not significant, suggesting that the talent policies enhance the number of citations of patents with a long-term effect, which remains valid at least from the 4th to the 5th year after policy implementation.

**Table 9:** Quantile regression results.

Independent variables	(1)	(2)	(3)	(4)	(5)	(6)	(7)
	Current period	Lag 1 period	Lag 2 period	Lag 3 period	Lag 4 period	Lag 5 period	Lag 6 period
PC-99	0.00026	0.009	-0.033	-0.007	-0.009	-0.046	-0.045
t-Value	0.00	0.16	-0.51	-0.10	-0.11	-0.54	-0.43
PC-95	0.053^***^	0.048^**^	0.049^**^	0.046^*^	0.054^**^	0.059^**^	-0.006
t-Value	2.70	2.48	2.32	1.83	2.08	1.95	-0.14
PC-90	0.068^***^	0.066^***^	0.054^***^	0.051^***^	0.048^**^	0.018	0.005
t-Value	4.69	4.41	3.33	3.02	2.54	0.80	0.20
PC-85	0.076^***^	0.079^***^	0.066^***^	0.061^***^	0.065^***^	0.035^*^	0.028
t-Value	5.76	5.50	4.23	3.79	3.69	1.68	1.25
PC-80	0.061^***^	0.066^***^	0.063^***^	0.051^***^	0.051^***^	0.029^*^	0.023
t-Value	5.62	5.81	4.99	3.65	3.27	1.73	1.21
PC-75	0.059^***^	0.056^***^	0.052^***^	0.045^***^	0.044^***^	0.015	0.002
t-Value	5.80	4.90	4.30	3.61	3.13	0.92	0.13

* ,

** , and

*** indicate significance at the 10%, 5%, and 1% levels, respectively.

From the perspective of different percentiles, the talent policies have the most significant impact on the number of citations of patents at the 85th percentile, showing a trend of first rising and then declining. For the percentiles with significant estimated coefficients, except for the 95th percentile, which shows a steadily increasing trend, the other percentiles display an overall declining trend. A comparison of the estimated coefficients at the 5th lag and the 1st lag shows that, between the 90th and 75th percentiles (PC-90 to PC-75), the effect of the talent policies decreases by between 55.696% and 73.214%. Overall, these findings validate the promoting effect of science and technology talent policies on key core technologies R&D.

### 5.5. Handling reverse causality and endogeneity issues

The science and technology talent policies implemented by governments at various levels in China are based on competitive selection processes, where applicants are evaluated according to their previous research performance. This implies that applicants may have already held key core technologies patents before receiving support from the talent policies, which introduces the potential for reverse causality. Furthermore, there may be unconsidered or difficult-to-quantify factors that influence the implementation of the talent policies, leading to endogeneity issues. Therefore, it is necessary to construct appropriate instrumental variables to address these two concerns. The suitable instrumental variables should be tested for over-identification and weak instruments, and we construct two instrumental variables for cross-validation.

The construction of instrumental variables aims to ensure that they are highly correlated with the science and technology talent policies and uncorrelated with other variables. These instrumental variables are then used in place of the talent policies variable for regression analysis. We argue that the number and quality of talent in an enterprise determine whether the enterprise can receive support from the talent policies, and the ability of an enterprise to recruit such talent is influenced by the city in which the enterprise is located. Specifically, two instrumental variables are constructed: IV1 is the ratio of the number of internet broadband users to the resident population in the city, and IV2 is the ratio of the city’s GDP to lottery sales. The rationale for selecting these two instrumental variables is as follows: First, using city-level data instead of enterprise-level data helps mitigate endogeneity issues. The specific indicators in these two instrumental variables are not directly correlated with the R&D output of the sample enterprises, nor with other variables that influence R&D output. Even if correlations exist, they are likely to be very weak. For instance, the number of internet broadband users and GDP may include data from sample enterprises located in the city, but the weight of these enterprises in the overall city-level indicators is negligible, which ensures the external validity of the instrumental variables. Second, these two instrumental variables have a logical relationship with a enterprise’s access to science and technology talent policies. IV1, the ratio of the number of internet broadband users to the resident population in the city, represents the city’s level of informatization and its diffusion. Since talent in R&D requires substantial access to knowledge and information, cities with higher levels of informatization and diffusion are more likely to attract talent. IV2, the ratio of the city’s GDP to lottery sales, represents the city’s institutional development level. The purchase of lottery tickets reflects speculative tendencies, and individuals with a higher level of speculation increase social transaction costs, such as defaults and non-compliance with regulations. This variable reflects the GDP produced by transaction costs, and higher institutional development may suppress speculation, thereby reducing transaction costs and making the city more attractive to talent. These two instrumental variables reflect that cities with higher levels of informatization and more developed institutions are more attractive to talent, thereby increasing enterprises’ ability to attract talent and facilitating their access to science and technology talent policies.

The results of the over-identification test are presented in Table 10. For both KCT1 and KCT2 indicators, the Score chi-squared values for IV1 and IV2 are not significant, and thus we cannot reject the null hypothesis of “all instrumental variables are exogenous”. This suggests that IV1 and IV2 are exogenous. We then proceed with the weak instrument test using the Wald test at a 5% nominal significance level. The minimum eigenvalue statistic for the two-stage least squares estimation is 12.704, which is greater than the critical value of 11.59 for the 10% significance level. Therefore, we can reject the null hypothesis of “weak instruments”, indicating that weak instruments are not present. The regression results using the instrumental variables are shown in Table 11. It is found that IV1 and IV2 have significant positive estimated coefficients for KCT1 and KCT2, respectively, which indicates that the estimated results for the impact of the science and technology talent policies on key core technologies R&D are robust.

**Table 10:** Overidentification test results.

Variables	Instrumental variables	Score chi2	P-value
KCT1	IV1, IV2	0.426	0.514
KCT2	IV1, IV2	0.262	0.609

**Table 11:** Instrumental variable regression results.

Variables	(1)	(2)	(3)	(4)
	KCT1	KCT2	KCT1	KCT2
IV1	0.059^**^	0.033^***^		
t-Value	2.020	3.140		
IV2			0.006^*^	0.003^**^
t-Value			1.740	2.510
CV	yes	yes	yes	yes
year	yes	yes	yes	yes
industry	yes	yes	yes	yes
C	-9.219^***^	-2.293^***^	-9.202^***^	-2.279^***^
t-Value	-12.100	-8.410	-12.080	-8.350
F	28.153	3.022	28.146	3.019
Prob	0.000	0.000	0.000	0.000
Adj R^2^	0.224	0.194	0.223	0.194

* ,

** , and

*** indicate significance at the 10%, 5%, and 1% levels, respectively.

### 5.6. Other robustness tests

First, we consider the potential interference of government subsidies and enterprise R&D expenditure on the effect of the science and technology talent policies. Government subsidies obviously increase the additional income of enterprises, and this income may affect key core technologies R&D. Furthermore, enterprise R&D expenditure is a direct source of funding for technological R&D, which may also impact key core technologies R&D. To separate these two factors, we include government subsidies (GS), which are the government subsidy amount minus the science and technology talent policies support, and enterprise R&D expenditure (RD) in model (1). The regression results are shown in Table 12. In columns (1) and (2), the estimated coefficients of TP, GS, and RD are all significant. Except for the fact that the estimated coefficient of GS on KCT1 is larger than that of TP, the estimated coefficients of GS and RD are all smaller than those of TP, indicating that the positive effect of TP on KCT is greater than that of GS and RD, and the estimation results are robust.

**Table 12:** Other robust regression results.

Variables	Considering the role of GS and RD		Replacing independent variable		Adding fixed effects at the city level	
	(1)	(2)	(3)	(4)	(5)	(6)
	KCT1	KCT2	KCT1	KCT2	KCT1	KCT2
TP	0.019^*^	0.011^***^			0.034^***^	0.017^***^
t-Value	1.760	2.600			2.750	3.860
GS	0.025^***^	0.006^***^				
t-Value	9.080	6.420				
RD	0.005^***^	0.002^***^				
t-Value	6.010	6.530				
TPF			0.006^***^	0.002^***^		
t-Value			4.240	4.20		
CV	yes	yes	yes	yes	yes	yes
year	yes	yes	yes	yes	yes	yes
industry	yes	yes	yes	yes		
city					yes	yes
C	-0.747^***^	-0.217^***^	-0.816^***^	-0.238^***^	-0.832^***^	-0.232^***^
t-Value	-2.570	-2.630	-2.600	-2.690	-2.630	-2.680
F	44.340	60.110	44.960	61.570	9.350	13.170
Prob	0.000	0.000	0.000	0.000	0.000	0.000
Adj R^2^	0.230	0.198	0.224	0.194	0.250	0.216

* ,

** , and

*** indicate significance at the 10%, 5%, and 1% levels, respectively.

Second, we replace the independent variable by using the amount of support from the science and technology talent policies (TPF) as a substitute for TP, and construct a panel regression model where TPF is the independent variable. The dependent variable, control variables, time fixed effects, and industry fixed effects are all the same as in model (1). The regression results are shown in Table 12. In columns (3) and (4), the estimated coefficients of TPF on KCT1 and KCT2 are significantly positive, indicating that the estimation results are robust.

Third, we change the fixed effects. Considering that each enterprise is located in a different city, it may be affected by the comprehensive factors of the city in which it is located. We replace the industry fixed effects with city fixed effects and add them to model (1). The regression results are shown in Table 12. In columns (5) and (6), the estimated coefficients of TP on KCT1 to KCT2 are all significantly positive, indicating that the estimation results are robust.

## 6. Heterogeneity analysis

In order to deepen the understanding of the impact of science and technology talent policies on key core technologies R&D, we further analyze the heterogeneous effects of these policies. We hypothesize that there may be differences between enterprises with different ownership structures, and thus divide the sample enterprises into two categories: state-owned enterprises and non-state-owned enterprises. We categorize the sample based on the ownership data collected, and apply model (1) for regression analysis. The results are presented in Table 13. In columns (1) and (2), the estimated coefficient for the impact of the science and technology talent policies on KCT1 is significantly negative for state-owned enterprises, while the estimated coefficient for KCT2 is insignificant but still negative. This suggests that the science and technology talent policies have a suppressive effect on the key core technologies R&D of state-owned enterprises. This may be related to the characteristics of policy support for state-owned enterprises and the limitations of their incentive mechanisms. State-owned enterprises often receive various forms of policy support from the government, such as funding and tax incentives, making the policy resources relatively abundant. However, excessive policy support may result in the enterprises failing to fully utilize the potential of each policy, leading to resource dispersion and inefficiency. Additionally, the incentive mechanisms in state-owned enterprises are relatively conservative and rigid, with compensation, promotion, and reward systems subject to institutional constraints, making it difficult to flexibly adjust in order to incentivize the innovative potential of talent. As a result, even with policy support, the motivation and innovation potential of talent may not be effectively stimulated. Furthermore, the decision-making levels in state-owned enterprises are numerous, and the implementation process is complicated. The enactment of policies is often constrained by procedures and approval processes, further diminishing the practical effect of talent policies on R&D.

**Table 13:** Regression results of enterprise property rights samples.

Variables	State-owned enterprises		Non-state-owned enterprises	
	(1)	(2)	(3)	(4)
	KCT1	KCT2	KCT1	KCT2
TP	-0.049^**^	-0.006	0.072^***^	0.023^***^
t-Value	-2.230	-0.830	5.720	4.490
CV	yes	yes	yes	yes
year	yes	yes	yes	yes
industry	yes	yes	yes	yes
C	-7.229^***^	-2.090^***^	-0.621^***^	-0.192^***^
t-Value	-5.490	-4.90	-2.780	-3.050
F	20.850	29.310	28.470	38.920
Prob	0.000	0.000	0.000	0.000
Adj R^2^	0.247	0.221	0.230	0.196

* ,

** , and

*** indicate significance at the 10%, 5%, and 1% levels, respectively.

In sharp contrast, in columns (3) and (4), the estimated coefficients for the impact of the science and technology talent policies on KCT are significantly positive for non-state-owned enterprises, indicating that the policy has a significant positive impact on key core technologies R&D in these enterprises. This could be attributed to the specific characteristics of policy support, decision-making, and incentive mechanisms in non-state-owned enterprises. Unlike state-owned enterprises, non-state-owned enterprises typically face fewer policy supports and therefore place greater emphasis on the implementation of each policy, striving to maximize its utility. The decision-making process in non-state-owned enterprises is shorter, and their execution efficiency is higher, enabling them to rapidly convert the science and technology talent policies into practical support measures. Moreover, the incentive mechanisms in non-state-owned enterprises are more flexible, effectively attracting and motivating talent, thus stimulating their R&D drive. Talent policies in non-state-owned enterprises not only provide funding and resource support for talent but also enhance motivation by improving benefits such as children’s education, social security, government rewards, and the optimization of research team construction. Therefore, although non-state-owned enterprises receive less policy support, the efficiency of their execution mechanisms and flexibility in incentive systems make the effect of science and technology talent policies on key core technologies R&D more pronounced.

Additionally, R&D investment is an important factor influencing a enterprise’s R&D activities and the resulting outcomes. However, there is no unified standard for categorizing the amount of R&D investment in both academia and the business world. Based on the R&D investment ratios of the top 500 Chinese enterprises and the top 500 global enterprises, we define companies with an R&D-to-revenue ratio of 5% or more as high R&D investment enterprises, and those with a ratio lower than 5% as low R&D investment enterprises. The regression results are presented in Table 14. In columns (1) and (2), the estimated coefficients for the impact of the science and technology talent policies on KCT are significantly positive for high R&D investment enterprises, indicating that the policies have significant positive impact on key core technologies R&D in these enterprises. This may be because these enterprises typically have sufficient R&D funding and resources, place greater emphasis on technological innovation, and are therefore more willing to leverage talent policies to support their R&D activities. High R&D investment enterprises generally have larger-scale R&D activities with clear objectives, and the science and technology talent policies can provide additional funding, resources, and incentives, effectively promoting the innovation drive and technological breakthroughs of their R&D personnel.

**Table 14:** Regression results of enterprise R&D investment samples.

Variables	Enterprises with R&D investment greater than 5%		Enterprises with R&D investment less than 5%	
	(1)	(2)	(3)	(4)
	KCT1	KCT2	KCT1	KCT2
TP	0.047^***^	0.019^***^	-0.016	-0.001
t-Value	3.000	3.450	-1.000	-0.070
CV	yes	yes	yes	yes
year	yes	yes	yes	yes
industry	yes	yes	yes	yes
C	-0.497^**^	-0.125^**^	-1.896	-0.651
t-Value	-2.110	-2.210	-1.250	-1.240
F	33.320	44.280	16.550	23.590
Prob	0.000	0.000	0.000	0.000
Adj R^2^	0.234	0.199	0.229	0.204

* ,

** , and

*** indicate significance at the 10%, 5%, and 1% levels, respectively.

In sharp contrast, in columns (3) and (4), the estimated coefficients for the impact of the science and technology talent policies on KCT are insignificant for low R&D investment enterprises, indicating that the policies do not promote key core technologies R&D in these enterprises. This could be due to the relatively low R&D investment in such enterprises, which often show a lower level of emphasis on technological innovation and lack sufficient R&D resources and funding. In this case, even with the support of the science and technology talent policies, the actual effect of the policy is difficult to fully realize, as the enterprises themselves lack sufficient investment and commitment to R&D. As a result, the policies fail to effectively stimulate innovation, leading to the lack of observable impact.

## 7. Crowding out effects analysis

Against the backdrop of increasingly fierce global competition in science and technology, the Chinese Government has implemented science and technology talent policies to help key core technologies R&D. However, despite years of policy implementation, some key core technologies have not yet achieved substantial breakthroughs. Besides the high difficulty of R&D itself, are there other possible influencing factors? This study tries to explore from the perspective of the possible crowding-out effect of general technologies R&D on key core technologies R&D. Baseline regression results show that science and technology talent policies has a significant promotion effect on key core technologies R&D, but has a stronger promotion effect on non-key core technologies R&D (NKP) and utility model R&D (UM). In order to further analyze whether general technologies R&D (NKP and UM) crowds out the resources of key core technologies R&D, we introduced NKP and UM variables and their interaction terms with TP in model (1) for regression respectively, and the results are shown in Table 15.

**Table 15:** Regression results of crowding out effects.

Variables	(1)	(2)	(3)	(4)	(5)	(6)	(7)	(8)
	KCT1	KCT2	KCT1	KCT2	KCT1	KCT2	KCT1	KCT2
TP	0.037^***^	0.008^**^	0.006	-0.016^***^	0.024^***^	0.012^***^	0.056^***^	0.015^***^
t-Value	4.500	2.240	0.750	-3.950	2.520	3.240	5.840	3.790
NKP1	0.005^***^		0.004^***^					
t-Value	90.240		68.48					
NKP2		0.092^***^		0.074^***^				
t-Value		33.720		23.500				
UM1					0.005^***^		0.007^***^	
t-Value					46.970		39.350	
UM2						0.063^***^		0.068^***^
t-Value						22.630		18.490
TP×NKP1			0.002^***^					
t-Value			14.800					
TP×NKP2				0.060^***^				
t-Value				11.220				
TP×UM1							-0.003^***^	
t-Value							-13.950	
TP×UM2								-0.011^**^
t-Value								-2.090
CV	yes	yes	yes	yes	yes	yes	yes	yes
year	yes	yes	yes	yes	yes	yes	yes	yes
industry	yes	yes	yes	yes	yes	yes	yes	yes
C	-0.186	-0.215^***^	-0.149	-0.203^***^	-0.396^***^	-0.139^**^	-0.419^***^	-0.140^**^
t-Value	-1.350	-3.630	-1.090	-3.440	-2.520	-2.310	-2.680	-2.320
F	259.080	93.230	261.540	94.430	129.420	80.010	131.920	78.740
Prob	0.000	0.000	0.000	0.000	0.000	0.000	0.000	0.000
Adj R^2^	0.458	0.232	0.465	0.237	0.296	0.206	0.304	0.206

* ,

** , and

*** indicate significance at the 10%, 5%, and 1% levels, respectively.

The results show that in columns (1) and (2), both TP and NKP have a significant positive effect on KCT. However, after adding the interaction term, the regression coefficient of TP changed from significantly positive to insignificant (column (3)) and even to significantly negative in column (4), while the regression coefficient of NKP remained significantly positive. This suggests that the increase in NKP may have weakened the promotion of KCT by TP to some extent, implying a potential crowding-out effect. Similarly, in columns (5) and (6), the effect of UM on KCT is significantly positive, as is the effect of TP, but in both columns (7) and (8), the interaction term is significantly negative. This indicates that both UM and TP are able to promote KCT without considering the effect of TP on UM, but after considering the effect of TP on UM, UM is able to significantly inhibit the role of TP. However, due to the limited supportive effect of UM on KCT, the crowding out effect of UM on KCT is not significant compared to NKP. It is worth noting, however, that UM does inhibit the facilitating effect of TP on KCT.

In reality, whether an enterprise carries out key core technologies R&D is influenced by multiple factors, among which profitability, input cost and R&D risk are the core decision-making factors. General technologies R&D is less costly and more successful than key core technologies R&D, so enterprises are more likely to prioritize general technologies R&D under the condition of R&D financial constraints. In addition, the science and technology talent policies will set specific performance appraisal indicators, and talents need to accomplish the set goals within a limited time, thus tend to choose the R&D tasks that are easier to achieve. This appraisal pressure, combined with financial constraints, may lead to a crowding-out effect, as talents are more inclined to general technologies R&D rather than key core technologies R&D.

## 8. Conclusion and discussion

Existing studies generally focuses on the driving role of R&D funding in technological innovation, particularly in the context of government fiscal subsidies, tax reductions, and technology innovation projects [2–4]. These studies confirm the significant role of financial investment in promoting innovation in general technology fields, yet little attention has been paid to key core technologies. Furthermore, there is limited focus on the role of talent, which is crucial in technological R&D. To address this gap, this study uses a sample of 1363 listed enterprises in China from 2011 to 2023 to analyze the impact of science and technology talent policies on key core technologies R&D. The findings indicate that science and technology talent policies can promote key core technologies R&D, but their impact is more significant for non-key core technologies. This may be related to the higher difficulty of key core technologies R&D. Under performance evaluation goals, talent tends to focus on technologies that can produce faster research outcomes. This discovery offers a new perspective to the existing literature, highlighting the heterogeneous effects of science and technology talent policies across different technology fields with varying levels of R&D difficulty.

Regarding the analysis of different intensities and types of science and technology talent policies, this study finds that the stronger the support provided by science and technology talent policies, the greater their positive impact on key core technologies R&D. However, compared to non-key core technologies, the impact of three types of policies support with varying intensities on key core technologies R&D is relatively limited. This contradicts the findings of existing studies, which suggests that excessive policy support may inhibit technological innovation and that the relationship between policy support and innovation follows an inverted U-shape [18]. While existing studies mainly focus on the impact of government subsidies, tax reductions, and other policies on general technological innovation [54,55], this study concentrates on the role of science and technology talent policies in supporting key core technologies. As the intensity of policy support increases, enterprises gain access to more innovation resources, especially high-level talent and knowledge required for technological R&D. Therefore, the role of these policies in promoting key core technologies R&D becomes more pronounced. Moreover, key core technologies R&D involves higher technical barriers and long-term investment requirements, which may necessitate more sustained and stable policy support. This differs from general technological innovation [56]. Additionally, this study further explores the impact of different policy types and finds that talent subsidies, innovation incentives, talent introduction, and high-level talent innovation support policies, although differing in focus, all provide substantial support for key core technologies R&D. The positive impact of these policies is particularly evident. At the same time, we further analyzed the interactions of different policy combinations, and the results show that while individual policies generally have a facilitating effect on key core technologies R&D, there are significant offsetting effects between some policy combinations. For example, except for the insignificant interaction effects of talent subsidies and innovation incentives, and talent subsidies and high-level talent innovation support, the interaction terms of other policy combinations are all significantly negative, indicating that in some cases, the superposition of policies will not lead to a stronger promotional effect, but may instead have a certain offsetting effect due to changes in the allocation of resources or incentive mechanisms. In addition, we find that the regression coefficients of each individual policy increase after the introduction of the interaction term, which suggests that the effects of some policies may be overestimated when the interactions are not taken into account. Therefore, when optimizing talent policies, it is necessary to comprehensively consider the interactions between different policies to avoid potential loss of policy effectiveness. In particular, the promotion effect of the high-level talent innovation support policy is significantly smaller than that of the other three types of policies. This finding prompts us to re-examine the role of high-level talents in the key core technologies R&D and to further optimize the support methods and contents of talent policies, which provides a new empirical basis for policy formulation.

The study also finds that science and technology talent policies have significant positive impact on the number of highly cited patents. However, the influence diminishes as the lag period increases, with the decline ranging from 8.475% to 28.283%. Science and technology talent policies also promote the number of citations of patents, and this effect persists until the 4th or 5th year after policy implementation. However, the impact follows a declining trend, with the decrease ranging from 55.696% to 73.214%. It is evident that, relative to the number of patents, the effect of science and technology talent policies on the number of citations of patents experiences a larger decay over time. This is primarily because the number of patents is directly influenced by short-term factors, such as policy incentives and resource investments, leading to quicker effects. In contrast, the number of citations of patents is more influenced by long-term factors, including technology application, industry recognition, technological updates, and market demand. This f45inding is highly innovative, as most studies tend to use patent quantity as a measure of innovation output [57] while neglecting the number of citations of patents as an important indicator of innovation quality. Although existing literature has also identified the lagged effect of policy on patent quantity [58,59], it has not thoroughly explored the decay pattern of this effect. This study, through quantitative analysis of the lagged effect and decay magnitude of the number of citations of patents, provides a more comprehensive and nuanced framework for policy impact assessment, shedding light on the long-term effects of science and technology talent policies and their heterogeneous impacts on patent quantity and quality. This enables a deeper understanding of the effects of science and technology talent policies on both the number and quality of patents.

The study finds that science and technology talent policies have suppressive effect on key core technologies R&D in state-owned enterprises, while significantly promoting R&D in non-state-owned enterprises. This is because state-owned enterprises tend to be inefficient in utilizing their abundant resources and usually lack the competitive organizational capabilities associated with innovation compared to private enterprises [60]. This study provides new evidence for existing studies from the perspective of science and technology talent policies and key core technologies R&D. Additionally, this study finds that science and technology talent policies significantly promote key core technologies R&D in high R&D investment enterprises, but have no significant effect on low R&D investment enterprises. Existing studies has pointed out that R&D subsidies can significantly boost innovation output through increased R&D investment [61], indicating that R&D investment influences the effect of policy interventions. However, this study further dissects the effect of science and technology talent policies across enterprises with different levels of R&D investment, clearly stating that high R&D investment enterprises are better able to leverage policies to achieve technological breakthroughs. In contrast, low R&D investment enterprises fail to derive the same benefits from policy support due to insufficient R&D capabilities. Thus, while this study verifies existing conclusions, it also highlights the heterogeneous impacts of ownership structure and R&D investment levels on the effect of science and technology talent policies, offering new empirical support for the precise design of related policies.

The study further found that general technologies R&D (NKP and UM) has a crowding-out effect on key core technologies R&D to a certain extent. This result suggests that under the appraisal constraints of science and technology talent policies, talents may be more inclined to engage in general technologies R&D, which is easy to achieve results in the short term, rather than key core technologies, which have higher long-term investment and higher risk of failure. This finding further expands the existing literature’s understanding of the impact mechanisms of science and technology talent policies. Existing studies have mostly focused on how policy promotes innovation [2] and viewed technological innovation as a whole [40] without digging deeper into the differences in the impacts of policies on different types of technological innovations, especially the potential crowding-out effects under resource constraints. The findings of this study suggest that when optimizing science and technology talent policies, we should avoid taking short-term research outputs as the assessment standard alone, and instead increase support for long-term key core technologies breakthroughs, and encourage enterprises and talents to take on high-risk, high-return key core technologies R&D, in order to reduce the crowding-out effect of general technologies R&D on it.

## 9. Policy recommendations

### 9.1. Strengthening the support intensity of science and technology talent policies

First, the government should prioritize support for strategic technological fields such as artificial intelligence, integrated circuits, high-level equipment, and biomedicine. By formulating targeted science and technology talent policies, the government can promote the concentration of talent in these critical fields. Second, the government should increase funding for key core technologies R&D, utilizing diversified channels such as special funds and venture capital, to ensure continuous support for research projects. By providing long-term and stable funding to talent, innovation capabilities can be enhanced, and breakthroughs in key core technologies can be promoted. Third, the government should strengthen support in areas such as professional title promotion, spousal employment, children’s education, and housing subsidies to improve the living conditions of talent. Strengthening these comprehensive supports can effectively alleviate the concerns of talent, ensuring that they can focus on key core technologies R&D.

### 9.2. Optimizing the synergistic effects of science and technology talent policies

First, the government should increase subsidies for talent in key core technologies fields, particularly for innovative technology projects, in order to reduce R&D costs for enterprises and incentivize more talent to engage in the research of key core technologies. Second, the government should design special innovation reward mechanisms, encouraging enterprises and research institutions to provide R&D incentives for talent engaged in key core technologies R&D. This would stimulate innovation motivation, improve R&D efficiency, and enhance the conversion rate of research results. Third, more flexible and efficient talent introduction policies should be established, particularly for attracting overseas talent by offering tax incentives, housing subsidies, and other supporting measures. This would draw high-level talent into key core technologies fields and address gaps in technological innovation. Fourth, special funds should be established to support high-level talent in conducting research projects in key core technologies fields, while providing policy advantages, including research facilities, interdisciplinary team collaboration, and other support, to promote technological breakthroughs and industrial applications.

### 9.3. Extending the duration of support for science and technology talent policies

First, science and technology talent policies should clearly support long-term, goal-oriented key core technologies fields, such as artificial intelligence, integrated circuits, high-level equipment, and biomedicine, ensuring alignment with national strategic objectives. By setting long-term priority areas for technological R&D, the government can promote deep research by talent in frontier fields. Second, the support period of these policies should be extended to five years or more to ensure the continuity of specific policies, such as talent subsidies, innovation incentives, talent introduction, and high-level talent innovation support. Providing stable policy support each year would encourage talent to invest more effort in long-term projects, ultimately achieving technological breakthroughs and innovation. Third, assessments should focus on technological progress and innovation capabilities rather than merely short-term outcomes. Stage-based evaluations can be established to periodically review the execution of research projects. By implementing a longer-term assessment mechanism, talent can be motivated to maintain sustained innovation efforts, while adjusting the policy support intensity according to the stage of research and development.

### 9.4. Implementing differentiated measures for science and technology talent policies

First, for non-state-owned enterprises, the support for science and technology talent policies should be strengthened. By aligning with the flexible research management systems of these enterprises, more policy incentives and financial support should be provided. Through these incentives, non-state-owned enterprises can be encouraged to increase R&D efforts in key core technologies fields and attract more talent to engage in technological development. Second, for state-owned enterprises, science and technology talent policies should avoid overconcentration of resources and instead optimize the management mechanisms for innovation, gradually dismantling institutional constraints on talent innovation, and unleashing the R&D potential of state-owned enterprises in key core technologies fields. Third, for enterprises with high R&D investment, the support of science and technology talent policies should be further increased by providing more favorable measures. Through policy guidance, these enterprises can be encouraged to concentrate their efforts on breakthrough developments in key core technologies and accelerate the conversion of technological innovations into practical results. Fourth, for enterprises with low R&D investment, science and technology talent policies should guide them to gradually increase their R&D investment, providing necessary financial and technical support to improve the basic R&D capacity of these enterprises. Policy incentives should help these enterprises cultivate talent and gradually unlock their innovation potential in technological R&D.

## Supporting information

S1 File Article data. (XLSX)
